# An Engineered Supramolecular Pathway for Enhanced Electrochemiluminescence

**DOI:** 10.1002/advs.78181

**Published:** 2026-10-06

**Authors:** Maria Vittoria Balli, Madhurima Jana, Chiara Capolungo, Patrick Gruhs, Pierre Picchetti, Giovanni Valenti, Frank Biedermann, Luca Prodi

**Affiliations:** ^1^ Department of Chemistry “Giacomo Ciamician” University of Bologna Bologna Italy; ^2^ National Interuniversity Consortium of Materials Science and Technology (INSTM) University of Bologna Research Unit (UdR Bologna) Bologna Italy; ^3^ Institute of Nanotechnology (INT) Karlsruhe Institute of Technology (KIT) Karlsruhe Germany; ^4^ IRCCS Azienda Ospedaliero‐Universitaria di Bologna Bologna Italy

**Keywords:** [Ru(bpy)3]2+, electrochemiluminescence, host‐guest systems, signal enhancement, small‐molecule detection, supramolecular chemistry

## Abstract

Electrochemiluminescence (ECL) underpins ultrasensitive clinical assays, yet current platforms are largely dominated by antibody‒antigen immunoassay formats, which are intrinsically difficult to translate to small‐molecule detection. The limited size of small molecules, together with the lack of multiple accessible epitopes, restricts the applicability of conventional sandwich architectures and calls for alternative recognition strategies capable of directly coupling molecular binding to ECL signal generation. Herein, we introduce a supramolecular ECL approach in which a recognition unit (cucurbit[7]uril) is covalently integrated with an ECL‐active luminophore (tris(2,2′‐bipyridyl)ruthenium(II), [Ru(bpy)_3_]^2+^) to create a compact, self‐contained, and recognition‐responsive emitter. This architecture provokes an intramolecular ECL generation pathway by preorganizing the protonated coreactant tri‐*n*‐propylamine (TPrA) inside the host, leading to a 162% increase in ECL intensity compared to the benchmark [Ru(bpy)_3_]^2+^/TPrA system, and modulating [Ru(bpy)_3_]^2+^ excited‐state properties through dynamic intramolecular host–guest interactions. Competitive binding of small molecules, including clinically relevant metabolites and hormones, perturbs this intramolecular equilibrium and reprograms both ECL mechanism and enhancement, enabling highly sensitive yet antibody‐free detection down to the nanomolar range in buffered media. Hence, our results establish a general supramolecular design principle for the first self‐contained, recognition‐responsive ECL emitter, opening avenues toward modular and host‒guest engineered platforms for chemically programmed ECL transduction.

## Graphical abstract

A supramolecular electrochemiluminescent (ECL) system is presented in which cucurbit[7]uril (CB7) is covalently linked to [Ru(bpy)_3_]^2+^, forming a recognition‐responsive emitter. Host‒guest preorganization of tri‐*n*‐propylamine enables intramolecular ECL, boosting intensity by 162% compared to the standard system. Competitive binding perturbs this equilibrium, enabling sensitive, antibody‐free detection of small molecules in the nanomolar range.

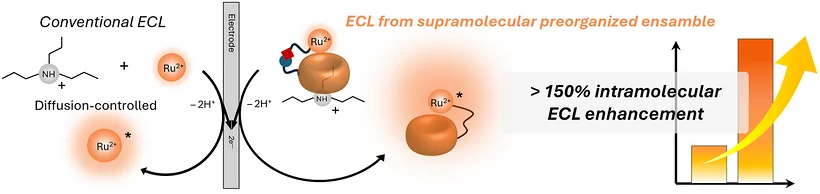

## 1 Introduction

The detection of small molecules is of broad relevance in diagnostics, environmental analysis, and food safety [1, 2, 3, 4, 5], encompassing targets such as metabolites, drugs, toxins, and pollutants. Yet, compared with proteins or nucleic acids, small molecules remain intrinsically more difficult to detect using conventional biosensing schemes, owing to their limited size, reduced structural complexity, and lack of multiple accessible epitopes. In this context, in the last 20 years, electrogenerated chemiluminescence (ECL) has undergone consistent evolutions, extending from classical systems [6, 7] toward more complex constructs endowed with molecular recognition capabilities. Characterised by the emission of light from a molecular species following an electron‐transfer reaction triggered by an electrochemical stimulus [8, 9], ECL has become a leading transduction technique for ultrasensitive bioanalysis [10], due to its high sensitivity, wide dynamic range, low background, and allowing the realization of highly cost‐effective and sensitive assays [11, 12, 13, 14]. Nowadays, this methodology has led to important commercial applications mainly focused on medical diagnostics: [15, 16, 17, 18, 19] the most successful example is represented by a ruthenium complex–based immunoassay analyser developed by Roche Diagnostics and Hitachi High Technologies [20, 21], i.e., the Elecsys technology. Traditionally, the most used ECL luminophore/coreactant pair is represented by the combination – in aqueous media – of tri‐*n*‐propylamine (TPrA) as coreactant and tris(2,2′‐bipyridyl)ruthenium(II) ([Ru(bpy)_3_]^2+^) as the ECL emitter. However, even though [Ru(bpy)_3_]^2+^ remains the benchmark dye in ECL, there is currently extensive research activity in developing new inorganic complexes, organometallic species or organic molecules [22, 23] able to yield high ECL intensities, either freely diffusing in solution [24] or embedded within nanosystems [25, 26, 27, 28], e.g., in silica nanoparticles [29], cucurbit[*n*]urils [30, 31], or cyclodextrins [32]. These systems are endowed with multifunctional features, such as specific molecular recognition capabilities, tuneable photophysical responses to external stimuli [32], and higher quantum yields [24, 29, 30]. However, the development of new ECL–active systems still remains a highly relevant topic, particularly in the context of enabling the design of stimuli‐responsive ECL emitters [33, 34]. For this reason, the exploration of novel systems integrating the ECL activity with supramolecular recognition motifs can open new perspectives to the field. In particular, a new class of dynamic and supramolecular ECL emitters (and thus novel signal generation pathways) can be unlocked. Moreover, since current *state‐of‐the‐art* strategies mainly rely on the use of costly and fragile antibody–based recognition elements or labelling [35, 36], introducing macrocyclic recognition elements offers a convenient and chemically more robust alternative for small organic molecules detection.

With this aim in mind, there is an extensive research centred on the investigation of dynamic binding interactions with small molecules of clinical interest, e.g., cellular metabolites or steroid hormones such as progesterone [37, 38, 39, 40, 41], able to modulate the photophysical properties of the dye, and consequently the ECL behavior [42]. Macrocyclic receptors – such as cyclodextrins [32, 43], calixarenes [44, 45], and cucurbit[*n*]urils (CB*n*) [29, 46, 47, 48] – have proven especially useful in this regard by providing means of supramolecular tethering of ECL‐active luminophores to either antibodies or electrode surfaces. Among these, CB*n* stand out due to their remarkable host–guest chemistry in aqueous solution [49], and CB7, in particular, exhibits binding affinities in the nanomolar to femtomolar [50] range for many small organic cations, enabling selective recognition even in complex biological or environmental matrices [51, 52]. Beyond simple host–guest binding, encapsulation within the CB7 cavity has been shown to significantly alter the physicochemical properties of luminescent guest molecules – modifying their absorption and emission spectra, stabilizing reactive intermediates, protecting fluorophores from quenching by dissolved oxygen or other reactive species, and increasing luminescence lifetimes [53, 54]. However, traditional applications of CB*n* rely on non‐covalent guest exchange and therefore are limited by equilibrium constraints, diffusion kinetics, and a lack of dynamic control over signal transduction mechanisms [55, 56, 57]. To address these limitations, we and others have demonstrated that conjugating CB*n* hosts with suitably chosen luminescent molecules enables the construction of unimolecular chemosensors and imaging agents [57, 58, 59, 60, 61, 62] combining the selective recognition properties of the macrocycle with the signal‐generating capabilities of the appended unit. While several luminescence–based chemosensors have been developed in this fashion, few systems have attempted to harness the potential of CB*n* in ECL–based detection platforms, especially the development of a signal‐enhanced ECL luminophore working directly in solution, without the need of performing any electrode surface functionalization or previous system capture, as commonly reported in the recent literature [29, 47, 48, 63, 64, 65, 66, 67].

Herein, as schematically depicted in Figure 1, we introduce a unimolecular, recognition‑responsive ECL emitter in which a cucurbit[7]uril (CB7) macrocycle is covalently tethered to a [Ru(bpy)_3_]^2+^–based ECL‐active system via a flexible tetraethylene‐glycol spacer, yielding a unimolecular construct (CB7–Rubpy, see Figure 1b). Unlike previously reported strategies, this design exploits host–guest preorganization of the coreactant TPrA within the CB7 cavity, in close proximity to the metal complex, to establish an unprecedented intramolecular ECL pathway in homogeneous solution. As a result, CB7–Rubpy delivers a remarkable 162% enhancement of the ECL signal compared to [Ru(bpy)_3_]^2+^, while simultaneously enabling reversible, analyte‑dependent modulation of the emission intensity, achieving more sensitive detection limits even for small molecules previously elusive to ECL detection, such as metabolites and drugs, without any electrode surface functionalization [46, 65, 68]. Furthermore, this design enables intramolecular association of the CB7 portals with the ruthenium complex itself, modulating the complex's photophysical properties and highlighting how dynamic supramolecular (intramolecular) interactions enable tuneable emission features, e.g., upon small‐molecule binding.

**FIGURE 1:**
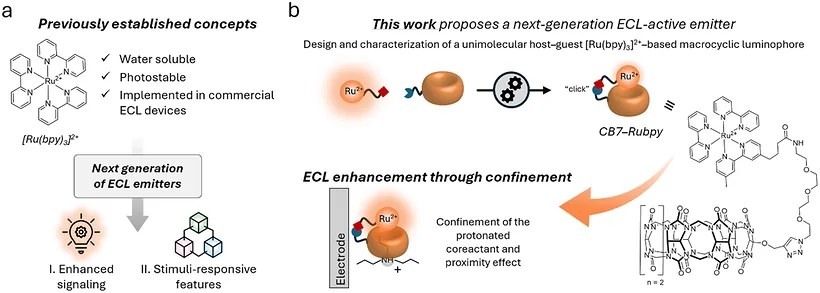
Schematic representation of [Ru(bpy)_3_]^2+^, its uses, and the herein presented work. (a) [Ru(bpy)_3_]^2+^ has long been a benchmark in ECL–based diagnostics, offering favorable physicochemical properties. Nevertheless, future diagnostic tools would benefit from the development of “next‐generation” ECL emitters that incorporate new strategies for signal enhancement and dynamic, stimuli‐responsive behavior, where luminescence properties can be modulated on demand in response to external chemical stimuli, e.g., presence of analytes. b) The work herein presented aims to design a unimolecular CB7–Rubpy ECL system (chemical structure reported) that enables tuneable luminescence properties of a [Ru(bpy)_3_]^2+^–based dye, while also introducing innovative means of ECL signal enhancement through host–guest interactions.

## 2 Results and Discussion

### 2.1 Design and Synthesis of the CB7–Rubpy System

As mentioned in the introduction, to surpass the current state of the art in ECL–active dyes, particularly regarding stimuli‐responsive features such as tuneable luminescence properties and novel strategies for signal amplification (see Figure 1), we first designed and synthesized a unimolecular CB7–Rubpy system (structure in Figure 1b) via a copper‐catalyzed azide–alkyne cycloaddition (CuAAC) [57, 58, 69] (see Figures S1–S3 for complete synthetic scheme and final product characterization) [70]. As we have already detailed in another manuscript [70], ^1^H–NMR spectra of CB7–Rubpy (Figure S4b) displayed a uniform upfield shift of ∼0.5 ppm for all the aromatic signals of the bipyridine ligand (with respect to the azide‐functionalized Ru(II) precursor, i.e., Ru–N_3_ in Figure S4a), as well as for the singlet corresponding to the methyl group on one of the bipyridine ligands (at 2.87 ppm), supporting the formation of a folded CB7–Rubpy conformation, in which the [Ru(bpy)_3_]^2+^ unit resides at the portal region of CB7. These observations, combined with ^1^H–NMR spectra upon addition to CB7–Rubpy of a high‐sterical hindrance guest, i.e., 1, 8‐octanediyl‐bis(methylimidazolium) ([C_8_(mim)_2_]^2+^, log *K*_a, CB7–NBD_ ≥ 8.0) [57], indicated both CB7–Rubpy ability to engage in host–guest binding and a significant displacement of the [Ru(bpy)_3_]^2+^–based moiety upon binding of larger guests (Figure S4c).

### 2.2 Photophysical and ECL Properties of CB7–Rubpy

Next, a detailed photophysical characterization was carried out for CB7–Rubpy, and its ability to bind high‐affinity guests was investigated. More in detail, absorption and emission spectra were recorded in comparison with a standard aqueous solution of [Ru(bpy)_3_]Cl_2_·6H_2_O: the absorption spectrum of CB7–Rubpy (Figure S5a) displayed an intense LC band in the UV region (290 nm) and moderately intense bands in the visible region (420–480 nm, ^3^MLCT), as it can be typically observed for Ru(II)–polypyridine complexes, although this latter band appears to be broader when the complex is linked to CB7. The photoluminescence quantum yield (QY, *Ф*) as well as the emission lifetime (*τ*) of CB7–Rubpy were determined in aqueous solution under air‐equilibrated conditions (*Ф*  =  0.012, *τ*_1_  =  92 ns – rel. % 12.1% – and *τ*_2_  =  275 ns – rel. % 87.9%). As also graphically shown in Figure S5b, c, the unimolecular system displayed a lower intensity and a clear biexponential lifetime decay in water, in contrast to the well‐known monoexponential decay of aqueous solutions of [Ru(bpy)_3_]^2+^ (*τ*  =  365 ns, *Ф*  =  0.028 in aerated water [71]). Furthermore, the phosphorescence peak of CB7–Rubpy (*λ*_max_  =  650 nm, Figure 2a) was slightly red‐shifted with respect to that of [Ru(bpy)_3_]^2+^ (*λ*_max_  =  622 nm).

**FIGURE 2:**
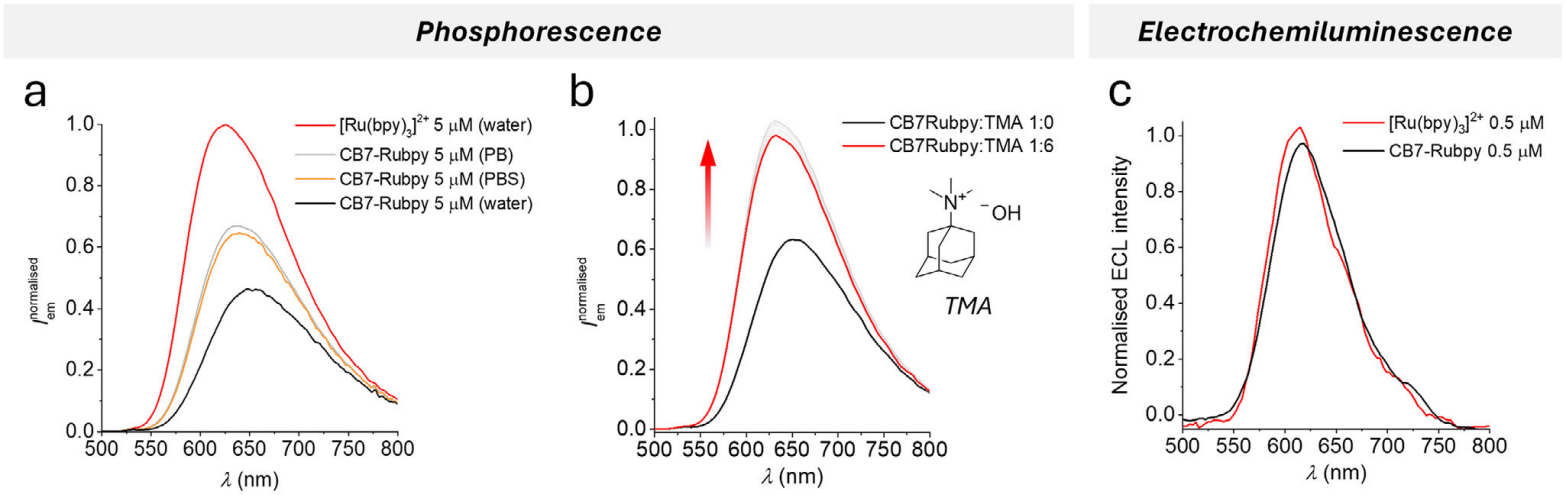
Emission properties of CB7–Rubpy under optical and electrochemical excitation. (a) Emission spectra of [Ru(bpy)_3_]^2+^ 5 µm (in water, red), and CB7–Rubpy 5 µm (in water, black; in PB 0.3 m pH 6.8, gray; in PBS 0.01 m pH 7.4, orange). (b) Emission spectra of a solution of CB7–Rubpy 5 µm upon addition of increasing amounts (1–6 eq.) of TMA in water. For the spectra of panels (a, b), ***λ***_exc_  =  450 nm, excitation and emission slits  =  10 nm, emission cut‐off  =  475 nm, normalized on the maximum emission intensity of the highest–response sample. (c) ECL spectra of [Ru(bpy)_3_]^2+^ 0.5 µm (red) and CB7–Rubpy 0.5 µm (black), recorded in PB 0.3 m at pH 6.8 in the presence of TPrA 180 mm. The two spectra are normalized each on its maximum emission intensity.

Interestingly, the lifetimes of CB7–Rubpy transform into a (longer) monoexponential decay upon changing the solvent, i.e., substituting water with PBS (0.01 m, pH 7.4, *τ*  =  304 ns) or PB (0.3 m, pH 6.8, *τ*  =  327 ns), being closer to the typical value of [Ru(bpy)_3_]^2+^ in the same conditions. At the same time, the phosphorescence of CB7–Rubpy (as from Figure 2a) gradually increases upon increasing the concentration (from 0.01 to 0.3 m) of the sodium cation, which is known to form relatively stable complexes with CB7 [49]. This enhancement was accompanied by a slight blue‐shift of the emission maximum, with *λ*_max, PB_  =  639 nm for CB7–Rubpy compared to *λ*_max, water_  =  650 nm (log *K*_a_ = 3.46) [47]. In line with this, when using a buffer in which Na^+^ and K^+^ cations are substituted with Li^+^ – which is known to show a much lower affinity for CB7 (log *K*_a, CB7_ = 2.34) – the photophysical properties of CB7–Rubpy are very similar to those observed in water, indicating that ionic environments weaken the interaction between CB7 portals and the Ru–moiety, and tend to restore native Ru‐polypyridyl photophysics.

All these findings can be explained by the formation of the intramolecular interaction between the reporter dye and the macrocycle to which it is linked, due to the partial intramolecular inclusion complex of the [Ru(bpy)_3_]^2+^–based moiety with CB7, in which the methylbipyridine unit is partially engulfed by the macrocycle (as indicated by the ^1^H–NMR data reported in the previous *Design and synthesis* section). Moreover, a similar behavior has also been observed when investigating a CB7–Rubpy solution (5 µm in water) in the presence or absence of a high‐affinity, spectroscopically transparent and electrochemically inactive guest, i.e., *N, N, N*‐trimethyladamantan‐1‐aminium hydroxide (TMA, log *K*_a, CB7_ = 12.2) [72]. As shown in Figure 2b, the addition of TMA to the CB7–Rubpy solution leads to an increase of the phosphorescence intensity (a behavior not observed upon addition of TMA to a solution containing only [Ru(bpy)_3_]^2+^, Figure S6b). All in all, these findings highlight the presence of an existing interaction between the Ru(II) moiety and the portal entrances of the CB7, interaction that can be either modulated or disrupted upon CB7 binding with a suitable guest. As a matter of fact, the previously reported sterically demanding guest [C_8_(mim)_2_]^2+^ is known to form with CB7 1:1 inclusion complexes in which both the imidazole units of the guest molecule protrude from the CB7 portals [73], thereby inducing a significant displacement of the [Ru(bpy)_3_]^2+^–based moiety. Similarly, TMA, although shorter, acts as a comparatively bulky guest for the macrocycle: due to the presence of the trimethylammonium group, complete inclusion within the CB7 is hindered, hence causing this portion of the guest to protrude from the macrocycle and again displace the Ru(II) moiety from the portal entrances [74]. Overall, the partial inclusion of the substituted bpy ligand of the [Ru(bpy)_3_]^2+^–based moiety in the CB7 cavity therefore modifies the electronic distribution on the complex, leading to a decrease of the energy of the ^3^MLCT excited state and – in line also with the energy‐gap law – to a decrease of the luminescence quantum yield and a shortening of the excited state lifetime. Moreover, a high concentration of cations in the aqueous buffer, which are well‐known to consistently interact with cucurbiturils [49, 57], leads to portal displacement of [Ru(bpy)_3_]^2+^, restoring the phosphorescence (single, longer lifetime).

With this in mind, the work hereafter presented aimed to further investigate whether the CB7 cavity could also be exploited for ECL applications, hypothesizing that the commonly employed coreactant TPrA might bind to the macrocyclic cavity. Particularly, since TPrA is an aliphatic amine, it holds the possibility to represent a suitable binding guest for CB7 in water. Hence, effective TPrA binding within the unimolecular system's cavity was expected to confine the coreactant in close proximity to the ECL‐active [Ru(bpy)_3_]^2+^–based luminophore and, upon electrical trigger, enable a more efficient ECL generation as a consequence of TPrA confinement, which would facilitate the homogeneous electron‐transfer process and the following signal generation. As a result, when a CB7–Rubpy solution (10 times diluted with respect to the photophysical experiments, 0.5 µm) is mixed with TPrA 180 mm, ECL emission can be triggered through a cyclic voltammetry (CV–ECL) scan (Figure S7a,d), even if working in buffer (PB 0.3 m, pH 6.8), as shown in Figure 3a. The ECL spectra of [Ru(bpy)_3_]^2+^ and the CB7–Rubpy system, reported in Figure 2c, are superimposable in position and shape, hence confirming that the emission originates in both cases from the same Ru‐centred ^3^MLCT state. To be noticed that, in this case, the ECL signal was consistently enhanced (162%) with respect to that of [Ru(bpy)_3_]^2+^ alone in the exact same conditions and concentration, thus providing a valuable tool for the signal enhancement in solution. The data shown in Figure 3a refer to the maximum of the ECL‐potential profile. Integrating instead the emission and the faradaic current over the same potential window, so as to obtain a charge‐normalized ECL output, CB7–Rubpy was found to emit two times more light per unit charge than [Ru(bpy)_3_]^2+^ under identical conditions (Table S1), with the integrated charge of the two systems differing by little more than 1%. Upon addition of 1 eq. of TMA, then, the ECL emission decreased, reaching a plateau value closer to the corresponding [Ru(bpy)_3_]^2+^ signal.

**FIGURE 3:**
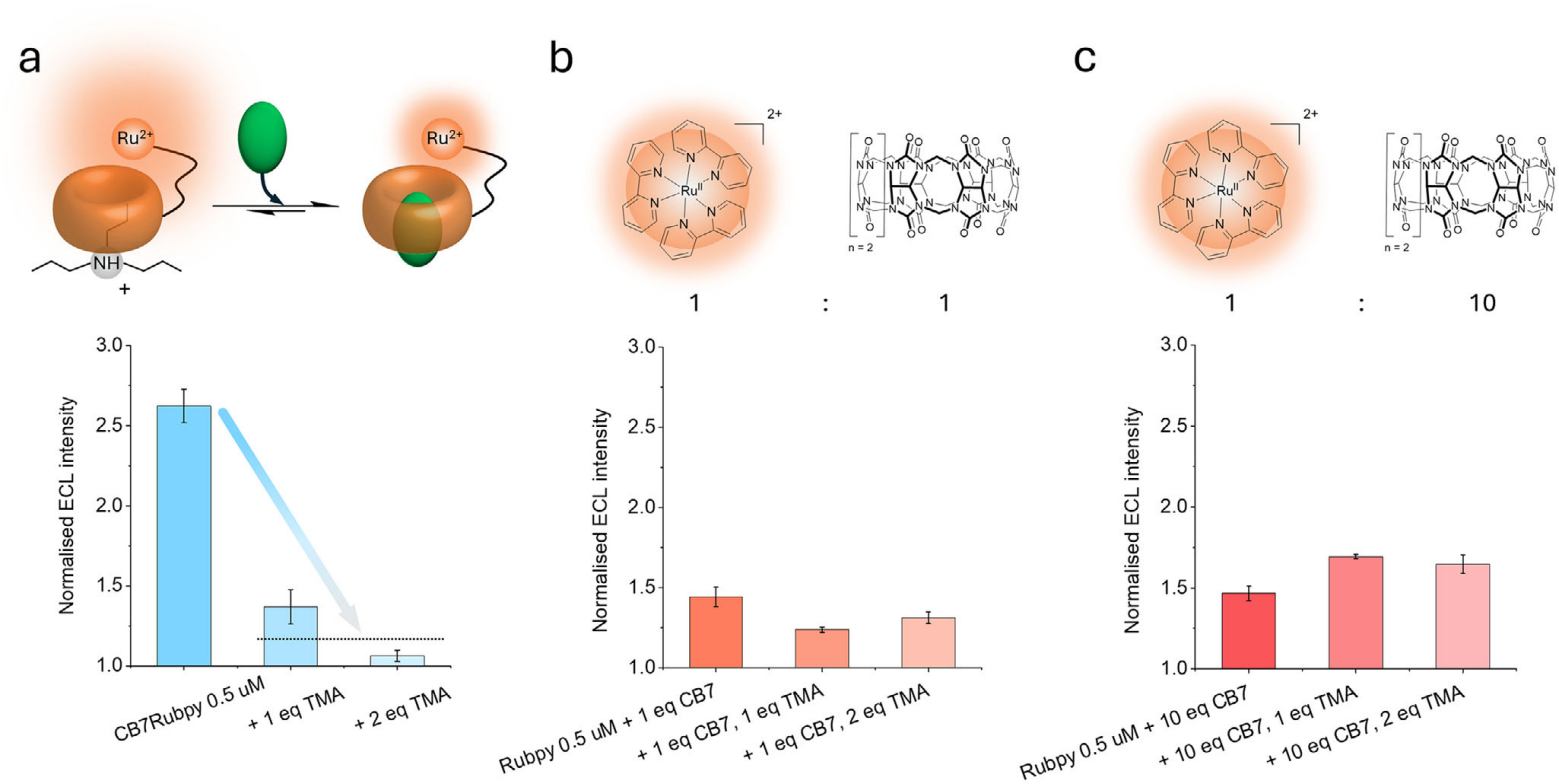
CV–ECL measurements performed in a 0.3 m PB solution at pH 6.8, with TPrA 180 mm. The working electrode potential was scanned at 100 mV/s, and emission acquired every 100 ms (control voltage 750 mV, photosensor module positioned on the short side of the cell). Amplification level at 00.0 µA. The resulting ECL signal intensity is normalized on the [Ru(bpy)_3_]^2+^ 0.5 µm ECL signal (which thus corresponds to the value of 1), and reported for (a) CB7–Rubpy 0.5 µm, either alone or in the presence of 1–2 eq. of TMA; b) [Ru(bpy)_3_]^2+^ 0.5 µm in the presence of 1 eq. of CB7, upon addition of 1–2 eq. of TMA; (c) [Ru(bpy)_3_]^2+^ 0.5 µm in the presence of 10 eq. of CB7, upon addition of 1–2 eq. of TMA (SD calculated for n ≥ 3). Corresponding non‐normalized ECL curves and CVs are reported in Figure S7.

The reported ECL working principle can be explained considering both the ^1^H–NMR spectra and the photoluminescent response of a CB7–Rubpy solution in the presence of TPrA. As shown in ^1^H–NMR reported in Figure S8 (enlarged on the TPrA behavior in Figure S9), TPrA was confirmed to bind to the CB7 moiety of the unimolecular CB7–Rubpy, in which TPrA signals experienced downfield shifts upon host–guest interaction. Specifically, this was true for the “a” and “b” protons, while the “c” ones remained largely unaffected. Furthermore, as shown by the photoluminescence response in Figure S10, it was again observed that portal displacement occurs upon the addition of TPrA (180 mm), a condition corresponding to the one used in a typical ECL experiment. Therefore, TPrA effectively interacts with the cucurbituril in a way similar to the one previously reported for TMA, but with a much smaller binding constant (log *K*_a, CB7–Rubpy_ = 3.98 for TPrA, as can be observed from the photoluminescent titrations and binding comparison reported in Figure S11). Moreover, CB7 possesses a single rigid hydrophobic cavity with an inner diameter of approximately 7.3 Å, portal diameters of approximately 5.4 Å, and a height of approximately 9.1 Å [52, 75], dimensions that are consistent with the inclusion of one suitably sized ammonium‐type guest at a time. Binary 1:1 inclusion complexes are indeed the established binding motif of CB7 with small cationic guests [51, 52, 75, 76]. In the present construct, this conclusion is further reinforced by the folded conformation of CB7–Rubpy, in which one of the two portals is engaged by the appended [Ru(bpy)_3_]^2+^ moiety (as from the ^1^H–NMR reported in the previous *Design and synthesis* section), leaving a single portal accessible to the coreactant.

Particularly, the best TPrA concentration for an effective ECL response was chosen upon comparing the behavior provided by adding to the CB7–Rubpy solution different TPrA amounts (20, 80, and 180 mm). Both the ECL intensity enhancement with respect to [Ru(bpy)_3_]^2+^ and the response to the addition of a competing guest (TMA) grow steadily with the coreactant concentration (Figures S12 and S13), while the decrease induced by 1 equivalent of TMA is not significant or remains within the experimental uncertainty at 20 and 80 mm (Figure S13). The 180 mm coreactant concentration was therefore selected for all the subsequent experiments, as the one providing both the largest enhancement and the most sensitive response to competitive binding. Furthermore, as from the photoluminescent response of CB7–Rubpy solutions in different solvents, reported in Figure S14, it is observed how the addition of TMA to the solution does not lead to phosphorescence increase anymore when working in TPrA 180 mm (and the response is strongly decreased as well in PBS and PB with respect to the one in water). Moreover, the consistent photoluminescent response reduction when working in the presence of TPrA is further confirmed by the average lifetimes trend (Figure 4), which are no longer affected by the addition of the high‐affinity guest, showing longer and monoexponential lifetimes (as indicated by the mean lifetimes values in Figure S15 ‒ complete decays extensively reported in Figure S16).

**FIGURE 4:**
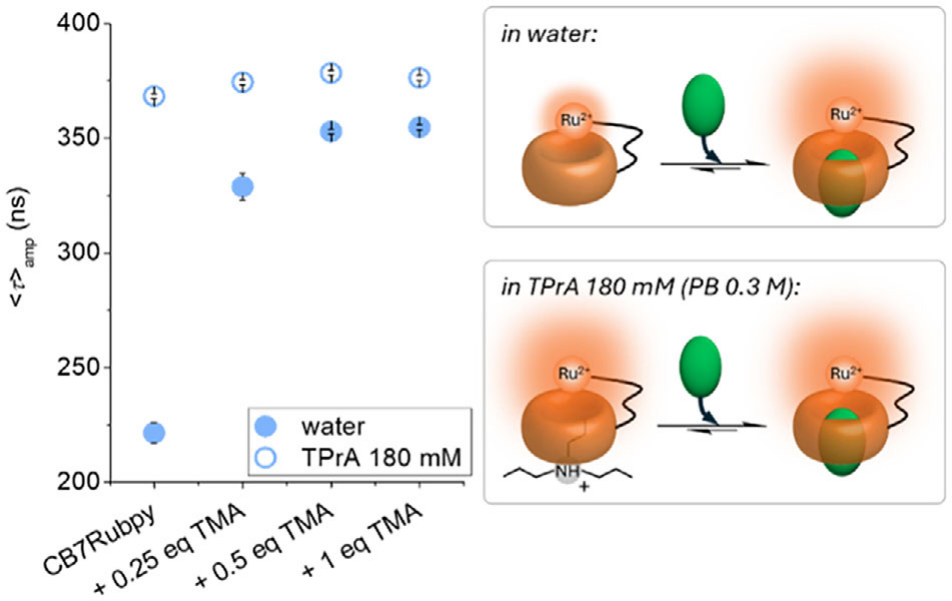
Mean values of < ***τ*** >_amp_, i.e., the average lifetime weighted on amplitude (SD calculated for n ≥ 3), of a solution of CB7–Rubpy 5 µm upon addition of increasing amounts (0.25–1 eq.) of TMA either in water (filled dots) or in the presence of TPrA 180 mm in PB 0.3 m pH 6.8 (empty dots).

The opposite response of CB7–Rubpy in phosphorescence (increase upon guest addition) or electrochemiluminescence (decrease upon guest addition) can be rationalised by considering the ability of TPrA to interact directly with the CB7 cavity. In the absence of competing guests, TPrA binds to the macrocyclic rim of the unimolecular construct and partially displaces the Ru(II) complex from the portal region, similarly to what is observed for TMA in purely photoluminescent experiments (Figure S11). Hence, the affinity effect of TPrA itself for cucurbiturils prevails over the [Ru(bpy)_3_]^2+^ quenching when dealing with ECL generation. Moreover, as evident from Figure 5, this host–guest interaction effectively confines the coreactant in close proximity to the ECL‐active [Ru(bpy)_3_]^2+^–based luminophore, promoting a pre‐organized, “intramolecular” environment in which the homogeneous electron‐transfer pathway between the oxidised coreactant and the metal complex is favored. It should be underlined that such a pre‐organization, i.e., CB7•TPrA association, is weak and highly dynamic, so that the complex is continuously formed, consumed and regenerated throughout the ECL experiment. On the basis of the association rate constants reported for CB7 with small cationic guests [77, 78, 79, 80], a vacated cavity is re‐occupied within microseconds at the coreactant concentration herein employed (TPrA 180 mm), the pre‐organized complex thus turning over several orders of magnitude more rapidly than the timescale of a CV–ECL scan, while persisting long enough for the ECL‐generating steps to occur.

**FIGURE 5:**
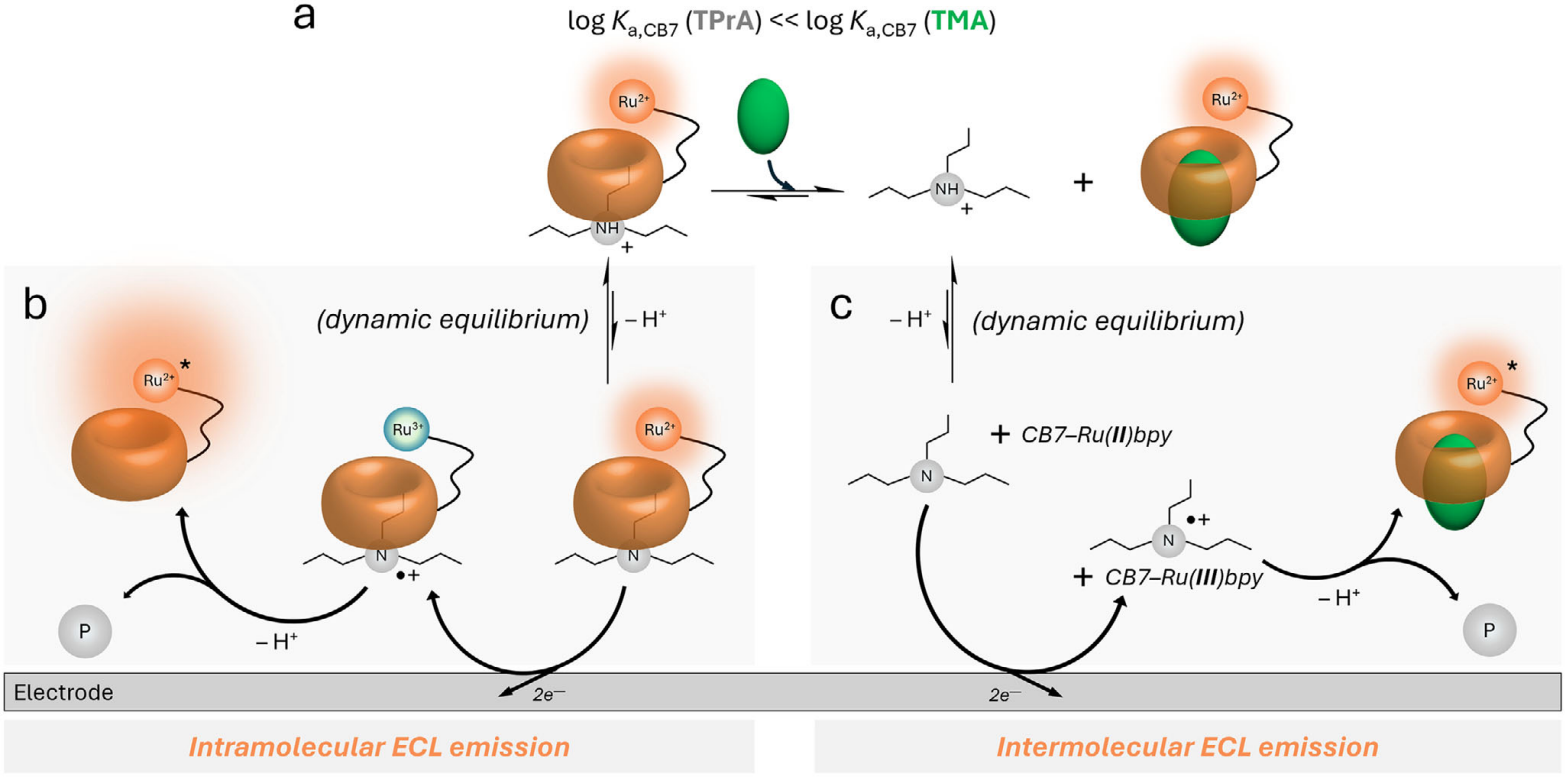
Schematic representation of the competitive intra‐ and intermolecular pathways leading to the ECL emission enhancement observed in the presence of the CB7–Rubpy unimolecular construct. The scheme depicts a single CB7–Rubpy unit; under the experimental conditions employed (CB7–Rubpy 0.5 µm, TPrA 180 mm) only a negligible fraction of the total coreactant resides within the macrocyclic cavity at any given time, so that the bulk speciation of TPrA is unaffected. (a) Competitive host–guest equilibrium at the CB7 portal. The protonated coreactant TPrAH^+^ is accommodated by the cavity of the macrocycle, in line with the well‐known preference of cucurbiturils for ammonium ions over neutral amines; addition of a higher‐affinity guest, e.g., TMA, displaces the coreactant and switches the system from the intramolecular to the intermolecular regime. (b) Intramolecular ECL pathway, operative in the absence of competing guests. The bound coreactant is in fast acid–base equilibrium with its neutral form, which is the species undergoing oxidation; one‐electron oxidation of the neutral amine and of the Ru(II) centre at the electrode generates, in close proximity to one another, the TPrA^•+^ and the CB7–Ru(III)bpy complex. Deprotonation of TPrA^•+^ yields the strongly reducing radical TPrA^•^, which reduces the Ru(III) centre to the emissive Ru(II)* excited state without requiring a diffusional encounter. (c) Intermolecular ECL pathway, operative when the cavity is occupied by a competing guest. The same sequence of steps takes place, but the coreactant and luminophore are no longer pre‐associated and the reduction of Ru(III) by TPrA^•^ relies on diffusion‐controlled encounters in bulk solution, resulting in a less efficient conversion of the radical intermediates into excited states. “P” denotes the finally resulting iminium cation, hydrolyzed in aqueous solution.

Consistent with the well‐known preference of cucurbiturils for ammonium ions over neutral amines [81, 82, 83], the CB7 cavity is expected to preferentially accommodate the protonated form of the coreactant, TPrAH^+^ (Figure 5a,b). Notably, acid‒base titrations carried out under the ECL conditions (Figure S17) show that the bulk protonation equilibrium of TPrA is not appreciably perturbed by the macrocycle, as anticipated from the vast sub‐stoichiometric excess of the coreactant with respect to the host: the preferential binding of TPrAH^+^ therefore operates locally, on the small fraction of coreactant residing within the cavity, without altering the overall speciation of TPrA in solution. As a result, the ECL process is significantly more efficient than in the case of freely diffusing mixing of [Ru(bpy)_3_]^2+^ and TPrA, yielding the marked enhancement in ECL intensity. This eliminates the diffusion contribution of the species for [Ru(bpy)_3_]^2+^* generation and provides a consistent enhancement of the ECL signal (Figure 3a) with respect to the case in which only CB7 and [Ru(bpy)_3_]^2+^ are mixed in solution (1:1 or 1:10 ratios, Figure 3b,c, respectively), without being connected through the unimolecular design, hence confirming that covalent linkage between the macrocycle and the luminophore is crucial to sustain the enhanced, intramolecular ECL pathway. It is worth noting that the modest enhancement observed upon simple addition of CB7 to [Ru(bpy)_3_]^2+^ cannot be ascribed to an association between the macrocycle and the luminophore: as we have already shown for this same system [70], free CB7 and free [Ru(bpy)_3_]^2+^ do not form an appreciable complex in water, neither ^1^H–NMR nor emission spectra being affected by the addition of the macrocycle. This residual contribution, which is significantly smaller than the one obtained with the unimolecular construct and is not suppressed by a competing guest, is therefore mechanistically distinct from the intramolecular pathway described here.

Upon addition of a high‐affinity CB7‐binding guest, e.g., TMA, which competes effectively with TPrA for the cucurbituril cavity (Figure 5a), the intramolecular pre‐organization is then disrupted. The macrocycle preferentially encapsulates the incoming guest, thereby expelling TPrA from the vicinity of the Ru(II) centre and shifting the system toward an “intermolecular” ECL regime, in which coreactant and luminophore once again rely on diffusion‐controlled encounters in bulk solution. Under these conditions, the proximity effect is lost, and the ECL signal decreases toward values comparable to those observed for [Ru(bpy)_3_]^2+^ alone (Figure 3a). This mechanistic picture is further corroborated by electrochemical data. As expected from the large excess of coreactant employed, the cyclic voltammograms recorded under all the investigated conditions are dominated by the oxidation of freely diffusing TPrA, and comparable currents and similar wave shapes are indeed observed in the presence of either the [Ru(bpy)_3_]^2+^/[Ru(bpy)_3_]^3+^ couple or the CB7–Rubpy system (Figures S18 and S19). Since the oxidised coreactant, and hence the amount of charge passed, is essentially unaffected by the nature of the luminophore, the pronounced differences in ECL intensity cannot be ascribed to a faster heterogeneous electron‐transfer: they must instead originate from the homogeneous reactions that follow it, whose efficiency is increased by the supramolecular pre‐organization of TPrA around the unimolecular CB7–Rubpy construct.

### 2.3 Further Application of the CB7–Rubpy ECL Enhancement

Eventually, we further provide a proof of concept for the application of CB7–Rubpy's ECL enhancement to the detection of some analytes of biological interest, i.e., nandrolone (NAND, a steroid hormone, log *K*_a, CB7_ = 7.05 in water) [84] and spermidine trihydrochloride (SPER, a biogenic amine, log *K*_a, CB7–TEG–BC_ = 4.49 in water) [58]. As it can be observed from Figure 6a, b and Figure S20, the unimolecular system can effectively be applied to the ECL detection of nandrolone and spermidine, decreasing the dye interaction with the CB7 cavity and, consequently, the related ECL signal intensity, following the shifting of the system toward the less efficient “intermolecular” regime. In line with these results, a similar behavior is also obtained for pancuronium bromide (PBr, log *K*_a, CB7_ = 5.1 for the 1:1 and log *K*_a, CB7_ = 10.2 for the 2:1 CB7:PBr complex) [59, 85], a steroid‐based neuromuscular blocking agent, which also induces a marked decrease of the ECL signal (Figure 6c). For NAND and PBr the signal does not change significantly on passing from 1 to 2 equivalents of analyte, remaining in both cases well below that of the analyte‐free system, whereas for SPER the decrease is resolved at each concentration step (Figure 6b). Hence, spermidine was selected for the quantitative estimation of its Limit of Detection (LoD) through ECL. As shown by the calibration plot reported in Figure 7, the LoD was calculated to be 76.44 nm (comparable to the value of 0.1 µm [86] typical of the enzyme–linked immunosorbent assays used for SPER detection, and significantly lower with respect to the 2.1 µm [87] recently obtained with a classic ratiometric fluorescent nanoprobe).

**FIGURE 6:**
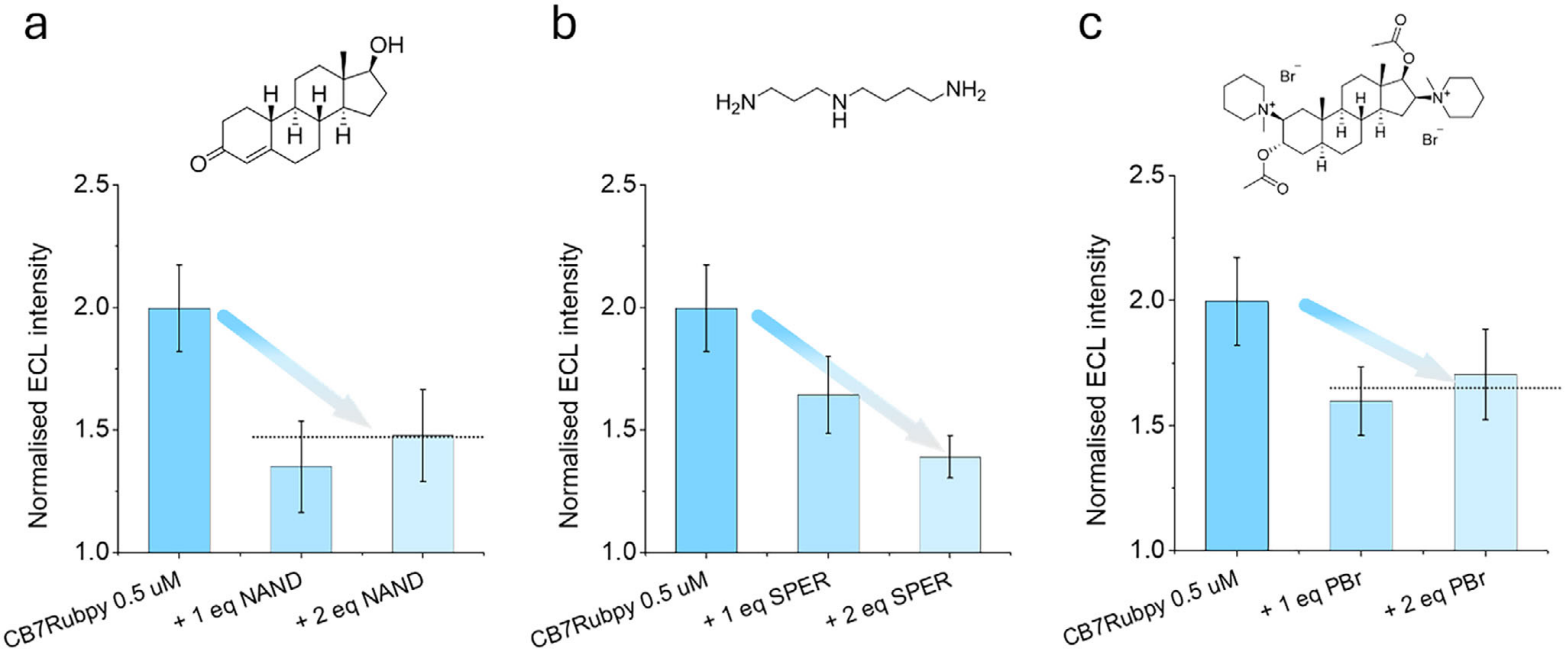
CV–ECL measurements performed in a 0.3 m PB solution at pH 6.8, with TPrA 180 mm. The working electrode potential was scanned at 100 mV/s, and emission acquired every 100 ms (control voltage 750 mV, photosensor module positioned on the short side of the cell). Amplification level at 00.0 µA. The resulting ECL signal intensity is normalized on the [Ru(bpy)_3_]^2+^ 0.5 µm ECL signal (which thus corresponds to the value of 1), and reported for a) CB7–Rubpy 0.5 µm, either alone or in the presence of 1–2 eq. of nandrolone; b) CB7–Rubpy 0.5 µm, either alone or in the presence of 1–2 eq. of spermidine trihydrochloride; c) CB7–Rubpy 0.5 µm, either alone or in the presence of 1–2 eq. of pancuronium bromide (SD calculated for n ≥ 3). Corresponding non‐normalized ECL curves and CVs are reported in Figure S20.

**FIGURE 7:**
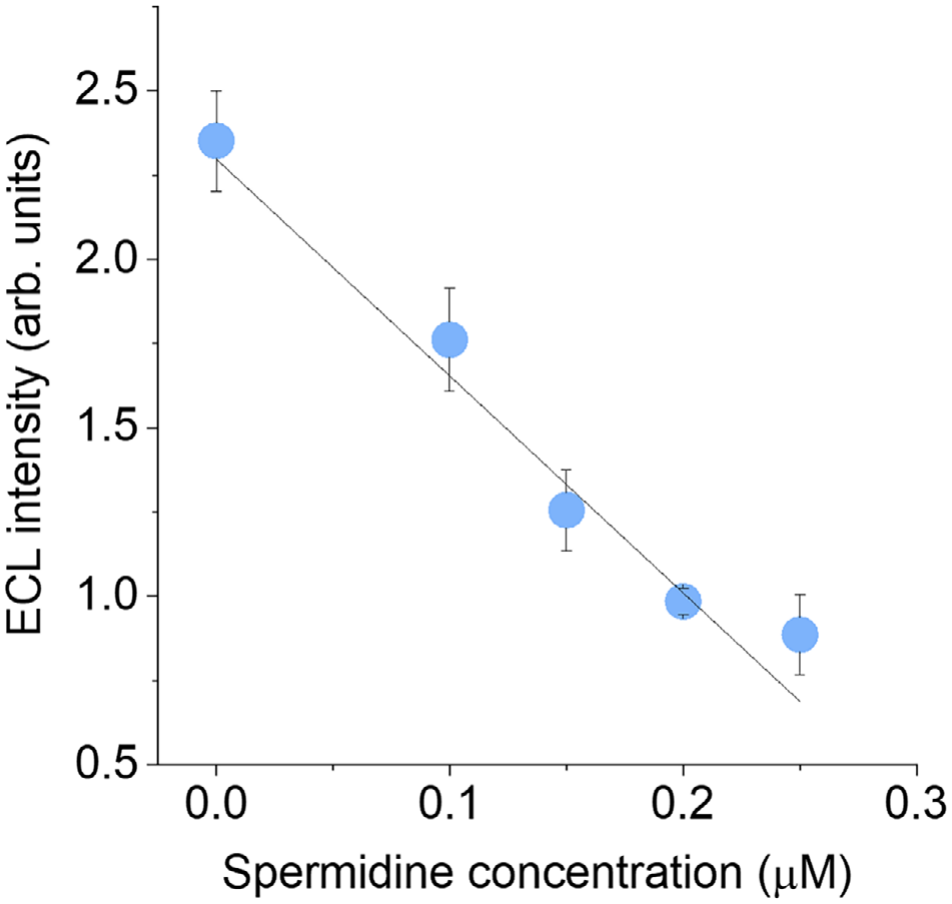
SPER calibration curve for LoD determination, reporting ECL maximum intensities for CB7–Rubpy 0.5 µm vs. the added spermidine trihydrochloride concentration (0–0.5 eq. SPER, SD calculated for n ≥ 3). Corresponding CVs and calibration equation are reported in Figure S21.

Therefore, we have demonstrated the ability of our unimolecular system to recognize small biogenic molecules, and in principle also steroid hormones, improving the overall ECL response, with the potential to further identify even different types of small metabolites through ECL changes, a task that has remained challenging with conventional antibody–based assays.

## 3 Conclusion

In summary, our work introduces a new molecular platform for dynamic modulation of photophysical and ECL properties through competitive binding principles. We have demonstrated that covalently integrating a cucurbituril host and a [Ru(bpy)_3_]^2+^‑type luminophore into a single unimolecular construct enables a new ECL generation regime, in which host–guest preorganization of the coreactant around the metal complex gives rise to intramolecularly enhanced ECL in homogeneous solution, resulting in unprecedented signal amplification without any electrode surface functionalization [46, 65, 68]. The unique supramolecular design results in a remarkable 162% enhancement of the ECL intensity compared to the benchmark [Ru(bpy)_3_]^2+^ system, even in buffered aqueous media, where classical unimolecular photoluminescent assays typically fail. This intrinsic structural synergy not only enhances the ECL performance but also introduces selective molecular recognition capabilities toward small biogenic amines and steroidal analytes, a task that has remained challenging with conventional antibody–based assays. The resulting cost‐effective and stable platform bridges supramolecular chemistry with ECL, unlocking new opportunities for label‐free and highly sensitive detection of molecular targets in complex biological environments. Overall, the results presented herein represent an important finding for the development of stimuli‐responsive and innovative ECL–based sensors, and they establish CB7–Rubpy as the first example of a self‐contained, recognition‐driven ECL platform, paving the way toward a new generation of smart, antibody‐free molecular sensors that couple sensitivity and operational simplicity within a single molecular architecture [69, 70].

## Author Contributions

**Maria Vittoria Balli**: conceptualization, data curation, formal analysis, investigation, methodology, visualization, writing – original draft, writing – review and editing. **Madhurima Jana**: data curation, investigation, methodology, writing – review and editing. **Chiara Capolungo**: methodology, investigation, writing – review and editing, data curation. **Patrick Gruhs**: data curation, methodology, investigation, writing – review and editing. **Pierre Picchetti**: conceptualization, formal analysis, investigation, methodology, visualization, writing – review and editing. **Giovanni Valenti**: conceptualization, resources, supervision, visualization, writing –review and editing. **Frank Biedermann**: conceptualization, resources, supervision, visualization, writing – review and editing. **Luca Prodi**: conceptualization, funding acquisition, resources, supervision, visualization, writing – review and editing, project administration.

## Conflicts of Interest

G.V. and L.P. are shareholders of Bionys s.r.l., a company active in ECL technology. The other authors declare no conflicts of interest.

## Supporting information




**Supporting File**: advs78181‐sup‐0001‐SuppMat.pdf.

## Data Availability

The data that support the findings of this study are available from the corresponding author upon reasonable request.
