# Supplementary material for: An Engineered Supramolecular Pathway for Enhanced Electrochemiluminescence
Source: Adv Sci (Weinh). 2026 Oct 6:e78181. Online ahead of print. doi: 10.1002/advs.78181 (PMC13643096; doi:10.1002/advs.78181)
Supplement: Supplementary file 1 [file ADVS-9999-e78181-s001.pdf]

Supporting Information

An Engineered Supramolecular Pathway for Enhanced Electrochemiluminescence

Maria Vittoria Balli,[a,b,+] Madhurima Jana,[a,+] Chiara Capolungo,[a] Patrick Gruhs,[c] Pierre Picchetti,[c] Giovanni Valenti,[a,*] Frank Biedermann,[c,*] Luca Prodi,[a,d,*]

[a]Department of Chemistry "Giacomo Ciamician", University of Bologna, via Gobetti 85, 40129 Bologna (Italy)

[b]National Interuniversity Consortium of Materials Science and Technology (INSTM), University of Bologna Research Unit (UdR Bologna), via Gobetti 85, 40129 Bologna (Italy)

[c]Institute of Nanotechnology, Karlsruhe Institute of Technology (KIT), Kaiserstrasse 12, 76131 Karlsruhe (Germany)
[d]IRCCS Azienda Ospedaliero-Universitaria di Bologna, via Albertoni 15, 40138 Bologna (Italy)

[+]These authors contributed equally.

**Correspondence to: g.valenti@unibo.it; frank.biedermann@kit.edu; luca.prodi@unibo.it*

Table of contents

1. List of variables and abbreviations ... 3

2. Materials and instrumentation ... 4

2.1 Chemicals ... *4*

2.2 Experimental ... *4*

2.2.1 Absorption and emission spectroscopy ... 4

2.2.1.1 Photoluminescence quantum yield ... 4

2.2.1.2 Experimental emission–based detection ... 5

2.2.2 Excited state lifetime decays ... 5

2.2.2.1 Decays collection and processing ... 5

2.2.3 Electrochemiluminescence (ECL) instrumentation ... 6

2.2.3.1 Experimental ECL–based detection ... 7

2.2.3.2 Determination of the ECL signal enhancement ... 7

2.2.3.3 Determination of the charge-normalised ECL output ... 7

2.2.3.4 Determination of the Limit of Detection ... 8

2.2.4 Nuclear magnetic resonance (NMR) spectroscopy ... 8

2.2.5 High performance liquid chromatography (HPLC) ... 8

2.2.6 Electronspray ionization mass spectrometry (ESI–MS) ... 8

2.2.7 Synthetic remarks ... 9

3. Supporting data and figures ... 11

4. References ... 22

1. List of variables and abbreviations

Bpy	2,2'-bipyridyne
$[C_8(mim)_2]^{2+}$	1,8-octanediyl-bis(methylimidazolium)
CB7	cucurbit[7]uril
CB7–OH	mono-hydroxylated CB7
CB7–OPr	mono-propargyloxylated CB7
CB7–Rubpy	cucurbit[7]uril–tris(2,2'-bipyridyl)ruthenium(II) unimolecular assay
^{13}C–NMR	carbon 13 nuclear magnetic resonance spectroscopy
CuAAC	copper-catalyzed azide-alkyne cycloaddition
CV	cyclic voltammetry
DCC	*N,N*-dicyclohexylcarbodiimide
ECL	electrochemiluminescence
^{1}H–NMR	proton nuclear magnetic resonance spectroscopy
HPLC	high performance liquid chromatography
HR-ESI-MS	high-resolution electrospray ionization mass spectrometry
K_a	binding affinity
λ	wavelength
LC	ligand-centred
LoD	Limit of Detection
^{3}MLCT	metal–to–ligand charge transfer
MW	molecular weight
NAND	nandrolone
NHS	*N*-hydroxysuccinimide
OCP	open circuit potential
PB	phosphate buffer
PBr	pancuronium bromide
PBS	phosphate buffer saline
PL	photoluminescence
PMMA	polymethyl–methacrylate
PMT	photomultiplier tube
PTFE	polytetrafluoroethylene
QY (Φ)	quantum yield
$[Ru(bpy)_3]^{2+}$	tris(2,2'-bipyridyl)ruthenium(II)
SD	standard deviation
SPE	screen-printed carbon electrodes
SPER	spermidine trihydrochloride
τ	lifetime
$<\tau>_{amp}$	average lifetime weighted on amplitude
TCSPC	time-correlated single photon counting
THPTA	tris(3-hydroxypropyltriazolyl)-methylamine
TMA	*N,N,N*-trimethyladamantan-1-aminium hydroxide
TPrA	tri-*n*-propylamine

2. Materials and instrumentation

2.1 Chemicals

Ultrapure (Milli-Q) water was used for all experiments. The PBS buffer (pH 7.4, 1X; 0.137 M NaCl, 0.0027 M KCl, 0.01 M Na_2HPO_4, and 0.0018 M KH_2PO_4) was prepared from Sigma-Aldrich PBS tablets. Tri-*n*-propylamine (TPrA, MW = 143.27 g/mol, ≥ 98% v/v), sodium phosphate monobasic dihydrate ($NaH_2PO_4 \cdot 2H_2O$, MW = 156.01 g/mol, ≥ 99%), sodium phosphate dibasic (Na_2HPO_4, MW = 141.96 g/mol, ≥ 99.5%), phosphoric acid (H_3PO_4, MW = 98.00 g/mol, ≥ 85%), silver hexafluorophosphate, copper sulfate, L-ascorbate and *cis*-bis(2,2'-bipyridine)dichlororuthenium(II) were purchased from Sigma-Aldrich. Tris(2,2'-bipyridyl)ruthenium(II) chloride hexahydrate ($[Ru(bpy)_3]Cl_2 \cdot 6H_2O$, MW = 591.72 g/mol) was purchased from Merck Life Science s.r.l. *N,N,N*-trimethyl-1-adamantylammonium hydroxide (25% in water), 4-(4'-methyl-[2,2'-bipyridin]-4-yl)butanoic acid, 11-azido-3,6,9-trioxaundecan-1-amine and tris(3-hydroxypropyltriazolylmethyl)amine (THPTA) were purchased from BLDpharm. 1-Adamantylamine (MW = 151.25 g/mol, 97%), nandrolone (or 19-nortestosterone, MW = 274.40 g/mol), spermidine trihydrochloride (MW = 254.63 g/mol, ≥ 99.5%), pancuronium bromide (MW = 732.67 g/mol), hydrochloric acid (HCl, MW = 36.46 g/mol, 37%, ACS reagent), and sodium hydroxide (NaOH, MW = 40.00 g/mol) were purchased from Merck Life Science s.r.l.

Cucurbit[7]uril (CB7),[88] mono-hydroxylated cucurbit[7]uril (CB7–OH)[89] and mono-propargyloxylated cucurbit[7]uril (CB7–OPr)[69] were prepared by following procedures reported in literature. For performing ECL experiments, CB7 was purchased from Merck Life Science s.r.l. *N*-Hydroxysuccinimide (NHS) and *N,N*-dicyclohexylcarbodiimide (DCC) were purchased from Acros Organics. For the final purification of CB7–Rubpy, MCI GEL® CHP20P Polymeric Adsorbent was used. Column packing and regeneration was performed as instructed by the manufacturer.

Screen-printed carbon electrodes (SPEs, model C110) were purchased from Metrohm DropSens.

2.2 Experimental

2.2.1 Absorption and emission spectroscopy

UV–Vis absorption spectra were recorded at 25°C by means of a double beam Perkin-Elmer Lambda–45 spectrophotometer. Emission spectra were recorded with a Perkin-Elmer LS–55 spectrofluorometer equipped with a gated photomultiplier with modified S5 response (for operation up to around 650 nm) and a red-sensitive R928 photomultiplier for operation up to 900 nm. Plastic (PMMA) cuvettes with optical path length of 1 cm were used. Detector response–based corrections have been further applied to the reported spectra, in order to acquire a more precise information about the luminescence peaks' location and intensity.

2.2.1.1 Photoluminescence quantum yield

Luminescence quantum yields (Φ, QY; uncertainty, ± 15%) were determined using $[Ru(bpy)_3]Cl_2 \cdot 6H_2O$ aqueous solution (aerated) as a reference (Φ = 0.028).[90] The following equation was used to determine the value of luminescence quantum yield:[91]

$$\Phi = \Phi_{ref} \frac{I_X}{I_{ref}} \frac{A_{ref}}{A_X} \left(\frac{n_X}{n_{ref}} \right)^2$$

where Φ_{ref} represents the photoluminescent QY of the reference compound; I_{ref} and I_X are the emission intensities of the reference and the sample, respectively; A_{ref} and A_X are the absorbance at the excitation wavelength (450 nm) of the reference and the sample; n_{ref} and n_X are the refractive indexes of the solvents.

2.2.1.2 Experimental emission–based detection

In a typical procedure, a stock solution of CB7–Rubpy (2 mM in water) was diluted to 5 μM (in the considered solvent) in a PMMA cuvette (optical path length = 1 cm), to which a solution of the analyte was added in increasing concentrations. For each addition, the cuvette was shaken and then let to equilibrate under static conditions for 2 minutes. Emission spectra were recorded upon excitation at 450 nm, corresponding to the ^{3}MLCT absorption band of the compound. The changes in emission intensity occurring around 620 nm were investigated (excitation and emission slits = 10 nm, emission cut-off = 475 nm).

2.2.2 Excited state lifetime decays

Lifetime decays were recorded with an Edinburgh FLS1000 equipped with an EPL – Picosecond Pulsed Diode Laser (λ_{exc} = 475 nm, pulses duration < 100 ps, repetition rates up to 20 MHz) and a standard detector photomultiplier tube (PMT) –980 in cooled housing (wavelength coverage of 185 nm to 900 nm, dark count rate of < 50 cps – cooled to -20°C). The instruments is also equipped with a time-correlated single photon counting (TCSPC) device (the detector is operated in single photon counting mode throughout all time ranges, with a detector response width of ~600 ps). The TCSPC is based on the probability that a single photon emitted by a luminescent sample is collected by a proper high sensitivity detector: this probability is statistically correlated with the variation of the concentration of the excited states in solution.

2.2.2.1 Decays collection and processing

Lifetime decays experimental data are processed using the software provided by the instrument manufacturer. If the decay profile, when represented on a logarithmic scale, exhibits a linear trend, a monoexponential regression can be applied. However, in many cases the decay is not strictly linear on a logarithmic scale, or the monoexponential regression fails to provide an adequate description of the data. The quality of a regression is primarily assessed through the statistical parameter χ^2, which quantifies the discrepancy between the experimental data points and the fitted curve. χ^2 can indeed be defined as:

$$\chi^2 = \sum_{k=1}^{n} \frac{[N(t_k) - N_C(t_k)]^2}{N(t_k)}$$

where $N(t_k)$ represents the measured fluorescence decay function at time t_k, $N_C(t_k)$ is the corresponding value from the calculated decay function, and n is the total number of data points. A χ^2 value of 1 indicates an excellent agreement between experimental and fitted data, while values that exceed approximately 1.2 suggest that the monoexponential model does not adequately describe the system.

When monoexponential fitting is insufficient, more complex models must be employed, depending on the characteristics of the system under investigation. The most common alternative involves representing the decay of the total emission intensity, $I(t)$, as the sum of two exponential functions:

$$I(t) = B_1 \cdot e^{\left(-\frac{t}{\tau_1}\right)} + B_2 \cdot e^{\left(-\frac{t}{\tau_2}\right)}$$

where τ_1 and τ_2 are the lifetimes, while B_1 and B_2 are the preexponential factors. The interpretation of these preexponential factors is system-dependent. If the system contains two distinct and independent luminescent species (for example, due to the presence of an impurity), τ_1 and τ_2 represent the lifetimes of the two species, while B_1 and B_2 correspond to their respective emission intensities at time t = 0. In this case, the preexponential factors depend on the quantum yields, emission intensities at the detection wavelength, and concentrations of the two species.

Even in systems with a single luminescent compound, multiexponential decays can arise. This may occur when the compound exists in different chemical environments (*e.g.*, with varying degrees of solvent shielding), in multiple conformational states (*e.g.*, aggregated *vs* non-aggregated), or when interacting differently with a quencher or non-emissive partner. In such cases, the preexponential factors provide information about the relative fractions of molecules in each environment. For systems exhibiting multiple lifetimes, an "average" lifetime is frequently reported. Two definitions are commonly employed: the amplitude-weighted average lifetime and the intensity-weighted average lifetime.

The amplitude average lifetime ($<\tau>_{amp}$) weights each lifetime component (τ_i) by its amplitude (B_i):

$$\langle\tau\rangle_{amp} = \frac{\sum_{i=1}^{n} B_i \cdot \tau_i}{\sum_{i=1}^{n} B_i}, \text{ with } \sum_{i=1}^{n} B_i = 1$$

This average is characteristic of the fluorophore under steady-state conditions and can be directly related to rate constants. For this reason, $<\tau>_{amp}$ is most appropriate when a single luminophore exhibits multiple lifetimes due to conformational heterogeneity or environmental influences.

On the other hand, the intensity average lifetime ($<\tau>_{int}$) weights each lifetime component (τ_i) by the intensity of that component ($B_i \cdot \tau_i$):

$$\langle\tau\rangle_{int} = \frac{\sum_{i=1}^{n} B_i \cdot {\tau_i}^2}{\sum_{i=1}^{n} B_i \cdot \tau_i}$$

This measure is particularly relevant for ensembles of emitters with intrinsically different emission properties, such as nanomaterials of varying sizes. Conceptually, $<\tau>_{int}$ represents the average lifetime of a collection of excited-state populations, where each lifetime is weighted by its contribution to the total fluorescence intensity. The predominance of longer lifetimes – that contribute disproportionately in the $<\tau>_{int}$ – reduces the influence of short-lived species and amplitude fluctuations, giving the metric an apparent robustness to changes in model complexity.

Further details on these topics have been extensively explored and described elsewhere.[91–93]

2.2.3 Electrochemiluminescence (ECL) instrumentation

For each sample measurement, a new screen-printed carbon electrode (SPE, C110) was used, of which only the working electrode was employed: the screen-printed auxiliary and silver pseudo-reference contacts of the strip were left unconnected. Main features of SPEs: working electrode material = carbon (diameter 0.40 cm, position closer to the end of the strip – designed for work in solution applications); substrate material = ceramic. Substrate size: length = 3.38 cm, width = 1.02 cm, thickness = 0.05 cm.
To record the ECL intensity, a PTFE homemade electrochemical cell was built using a Starna glass cell (type 3/G/20, path length = 2 cm) and operated in a conventional three-electrode configuration, comprising the SPE carbon working electrode, an external Pt counter and an external Ag/AgCl (3 M KCl) reference electrode, chosen so as to ensure a well-defined reference potential and a counter electrode of adequate surface area; all the potentials reported in this work refer to the Ag/AgCl electrode. The ECL

emission was triggered by an anodic potential sweep during cyclic voltammetry, controlled by a SP–300 potentiostat. The emitted light was collected by a Hamamatsu photosensor module H13543-20 operated by a C10709-C3 power supply unit, being set through a control voltage of 750 mV. The resulting photocurrent was measured with a Keithley Model 6485 Picoammeter (Keithley Instruments Inc., Ohio, United States) set to 00.0 μA amplification range. The system was enclosed in a homemade dark box to avoid interferences from external light.

Concerning the ECL spectra, they were acquired using a SpectraPro2300i spectrophotometer (Princeton Instruments) and the emitted light was collected below the electrochemical chamber, integrating the signal for 150 ms every 3 nm and applying a constant potential of 1.6 V *vs* Ag/AgCl using a 0.3 M PB solution containing 0.5 μM of the luminophore and 180 mM of TPrA.

2.2.3.1 Experimental ECL–based detection

In a typical procedure, a different sample solution was prepared for each measurement, upon mixing together TPrA 180 mM in PB 0.3 M (pH adjusted to 6.8 upon H_3PO_4 ≥ 85% addition), CB7–Rubpy, and increasing amounts of the analyte. The solution to be investigated (4.2 mL) was then added to a homemade electrochemical cell (built as from *paragraph 2.2.3*). The ECL signal intensity was obtained upon triggering the system with a cyclic voltammetry (CV) scan. More in detail, CV–ECL plots were collected by scanning the working electrode potential at 100 mV/s from open circuit potential (OCP) up to 2.5 V (*vs.* Ag/AgCl 3 M KCl), back to 0 V (*vs.* Ag/AgCl) and, eventually, terminating the cycle at OCP. The solution emission during CV–ECL measurements was acquired every 100 ms through a photosensor module whose control voltage was set to 750 mV, positioned on the short side of the cell. For each frame, the ECL intensity was plotted versus the applied potential. The background signal was eliminated by subtracting, for each frame, the average ECL intensity value of the background, retrieved in a region where emission is not present.
The bar plots of the normalised maximum ECL intensities were then obtained by extracting, for each sample, the value corresponding to the maximum of its ECL-potential curve (located at 1.3–1.4 V) and normalising it on the related maximum value of the $[Ru(bpy)_3]^{2+}$ 0.5 μM sample, acquired under the exact same conditions, within the same experimental session and on the same batch of screen-printed electrodes, so as to compensate for the electrode-to-electrode variability intrinsic to these disposable devices.

2.2.3.2 Determination of the ECL signal enhancement

The CB7–Rubpy ECL signal enhancement with respect to the standard $[Ru(bpy)_3]^{2+}$ (under the exact same conditions and concentration) was calculated using the following equation:

$$ECL\ enhancement\ (\%) = \frac{ECL_{CB7-Rubpy}^{max} - ECL_{Rubpy}^{max}}{ECL_{Rubpy}^{max}} \cdot 100$$

2.2.3.3 Determination of the charge-normalised ECL output

The $[Ru(bpy)_3]^{2+}$ and CB7–Rubpy charge-normalized ECL outputs were calculated from the CV–ECL measurements acquired. Since the potential is swept linearly, the scan rate v can be defined as:

$$v = \frac{dE}{dt}$$

so that the charge Q is:

$$Q = \frac{1}{v}\int i \cdot dE$$

and the integrated emission is:

$$\frac{1}{v}\int ECL\cdot dE$$

The scan rate cancels exactly (being identical for all measurements) and the ratio between the two systems is:

$$\frac{\int ECL\cdot dE}{\int i\cdot dE}$$

which represent the charge-normalized ECL output.

2.2.3.4 Determination of the Limit of Detection

The linear range of the ECL calibration curve was fitted with a linear regression curve, and the LoD calculated from three independent measurements for each concentration point, using the following equation:

$$LoD = \frac{3.3\cdot(\sigma_{CB7-Rubpy})}{|slope|}$$

2.2.4 Nuclear magnetic resonance (NMR) spectroscopy

Proton (^{1}H)– and carbon (^{13}C)–NMR spectra were recorded in deuterium oxide, chloroform–d_3 or DMSO–d_6, depending on the sample, on a Bruker Avance 400 spectrometer at 25°C. The ^{1}H–NMR and ^{13}C–NMR chemical shifts (δ) are given in ppm and refer to residual protons on the corresponding deuterated solvent.

2.2.5 High performance liquid chromatography (HPLC)

Analytical HPLC purifications were performed on an LC–2000Plus HPLC system equipped with a UV–2075 UV–Vis detector and a Kromasil 100 C18 5 µM LC column (250 × 4.6 mm, Agela) at a flow rate of 0.8 mL/min.

2.2.6 Electronspray ionization mass spectrometry (ESI–MS)

Spectra were recorded on a Orbitrap Exploris 240 (MM10525C) mass spectrometer (ThermoFisher Scientific) equipped with a HESI II probe. The instrument was calibrated in the *m/z* range 200–2000 for positive mode, using premixed calibration solutions (FlexMix-Thermo Scientific). Spray voltage, sheath gas and auxiliary gas pressure were used in the range of 1.75 V, for positive mode. The ion transfer tube temperature was set to 320°C. Samples were prepared as follows: samples were dissolved in a 10 mM aqueous solution of $[C_8(mim)_2]Br_2$ and diluted to 1 mL with MeOH. MeOH:H_2O (1:1 v/v) mixture was used as eluent. Injection of the samples was carried out with the aid of a Hamilton® syringe 725RN 500 µL at a flow rate of 25 µL/min, using a single syringe pump.

2.2.7 Synthetic remarks

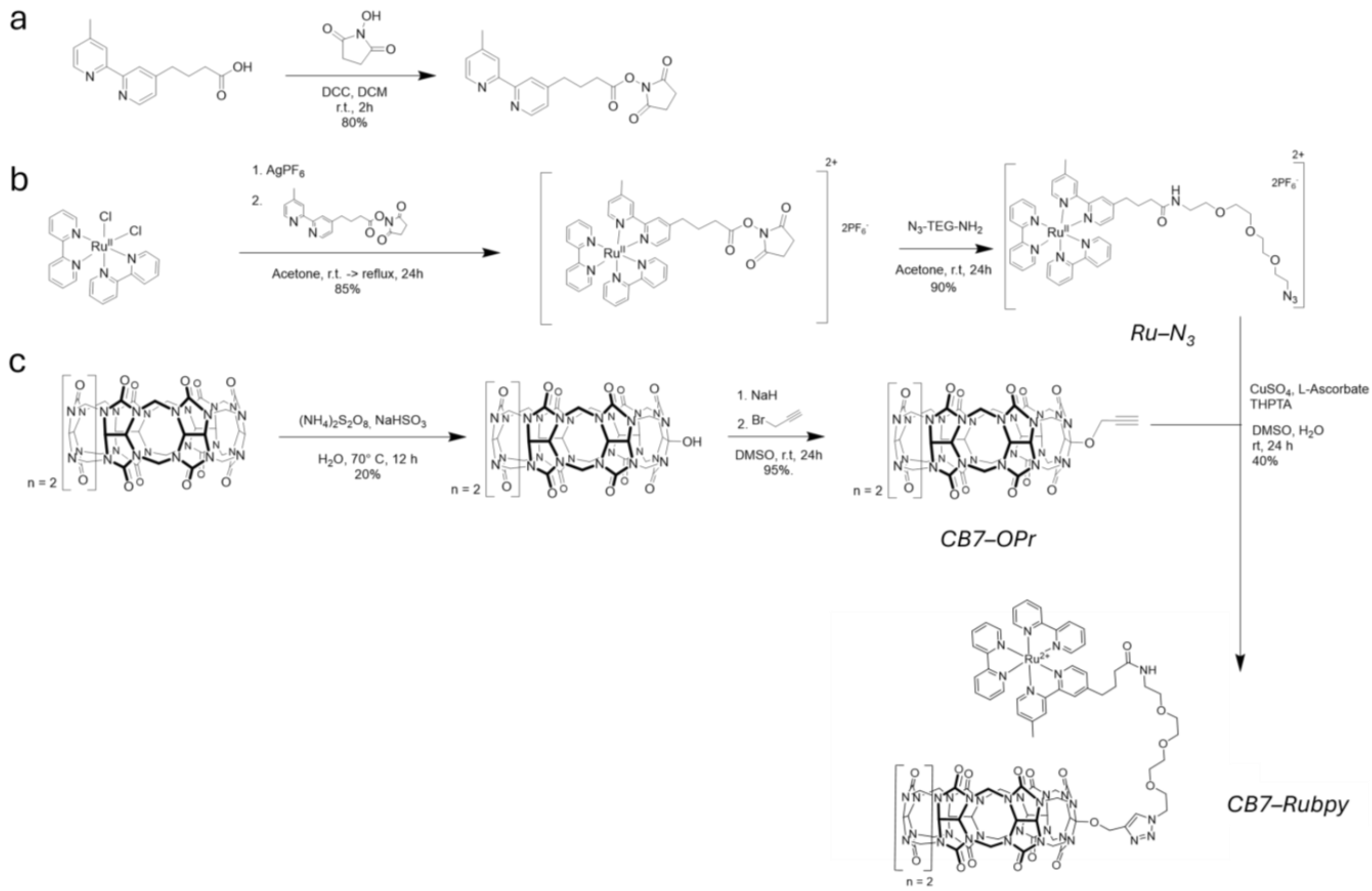

Fig. S1. *Schematic representation of the synthetic procedure for CB7–Rubpy, reporting the obtained yields for each step.*

The CB7–Rubpy system was synthesized *via* a copper-catalyzed azide-alkyne cycloaddition (CuAAC). More in detail, first of all the ruthenium precursor, *i.e.*, *cis*-bis(2,2'-bipyridine)dichlororuthenium(II), was converted into an azide–functionalized complex (Ru–N_3) through a three-steps sequence, involving its activation to the NHS ester (**Fig. S1a**) and the subsequent amidation with a bifunctional azido–amine linker (**Fig. S1b**). Subsequently, mono–propargyloxylated CB7 (CB7–OPr) was prepared from CB7 *via* sequential hydroxylation and *o*-alkylation. Eventually, the final click coupling of Ru–N_3 and CB7–OPr was carried out under standard CuAAC conditions in the presence of THPTA, producing the CB7–Rubpy in an isolated yield of 40% (**Fig. S1c**). The synthesis was reported as from ref.[70]

CB7–Rubpy

^{1}H–NMR (400 MHz, in D_2O) δ: 8.50 (d, J = 8.0 Hz, 4H), 8.36 (s, 2H), 8.10 (d, J = 11.2 Hz, 1H), 8.00 (t, J = 7.5 Hz, 4H), 7.79 (dd, J = 10.3, 5.4 Hz, 4H), 7.62 (dd, J = 18.1, 5.8 Hz, 2H), 7.34 (dd, J = 13.2, 7.1 Hz, 4H), 7.20 (dd, J = 10.5, 5.9 Hz, 2H), 5.99–5.33 (m, 26H), 4.47–4.14 (m, 14H), 3.91–3.82 (m, 2H), 3.48 (dq, J = 10.7, 5.5 Hz, 12H), 3.15–3.00 (m, 2H), 2.80 (t, J = 7.0 Hz, 2H), 2.64–2.55 (m, 4H), 2.48 (s, 3H), 2.27 (t, J = 7.0 Hz, 2H), 2.02–1.90 (m, 2H), 1.26–1.09 (m, 6H). Due to the complex structure, a precise assignment of all peaks was not possible. ESI-MS: calcd. for $[M+C_8(mim)_2]^{4+}$ = 590.7191; found = 590.7195. Analyt. HPLC: 4.98 min.

Additional information can be found in the Chemotion repository: https://complat-eln.ioc.kit.edu/mydb/collection/10651/reaction/61095.

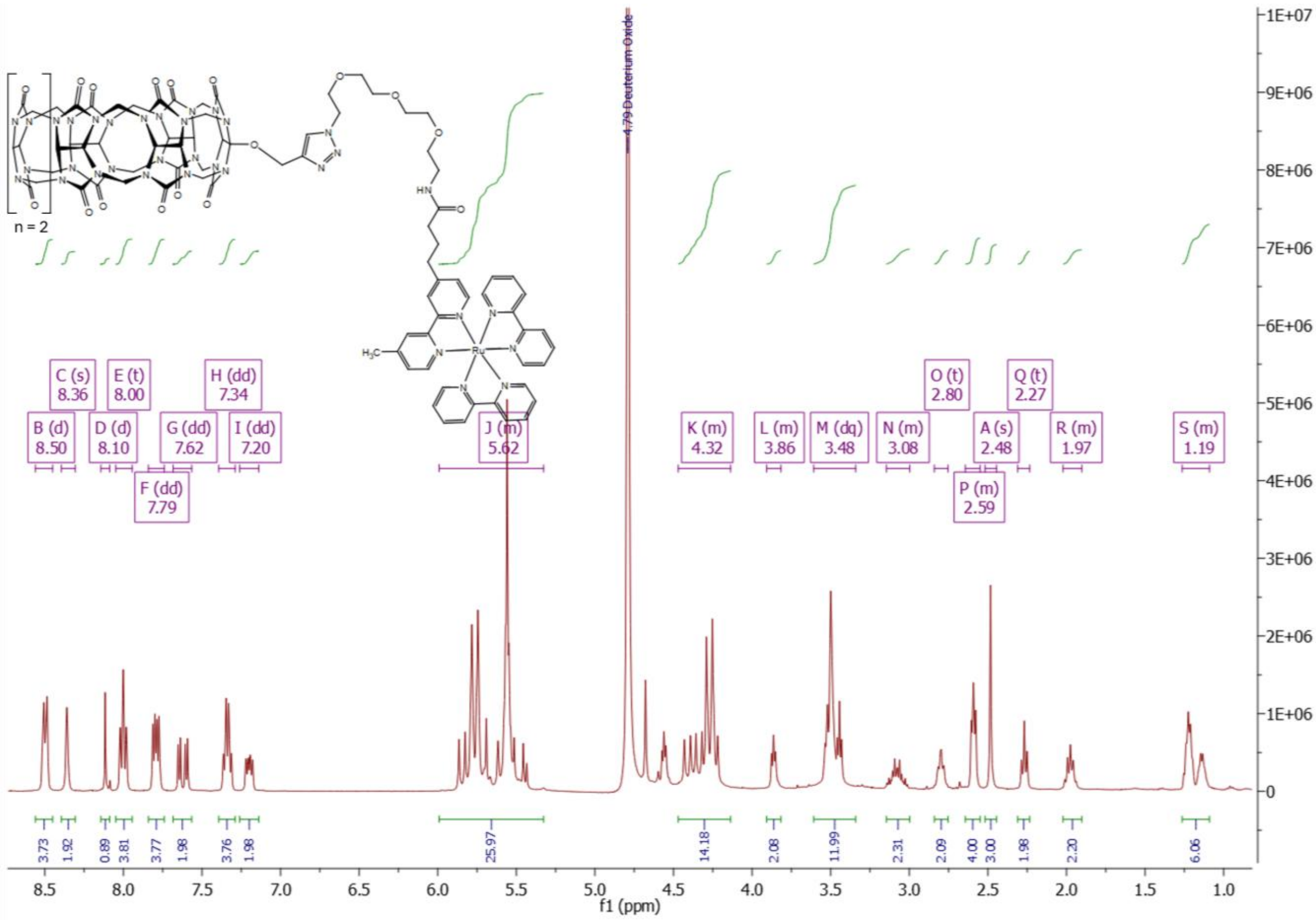

Fig. S2. *^{1}H–NMR of CB7–Rubpy 2 mM (400 MHz, in D_2O).*

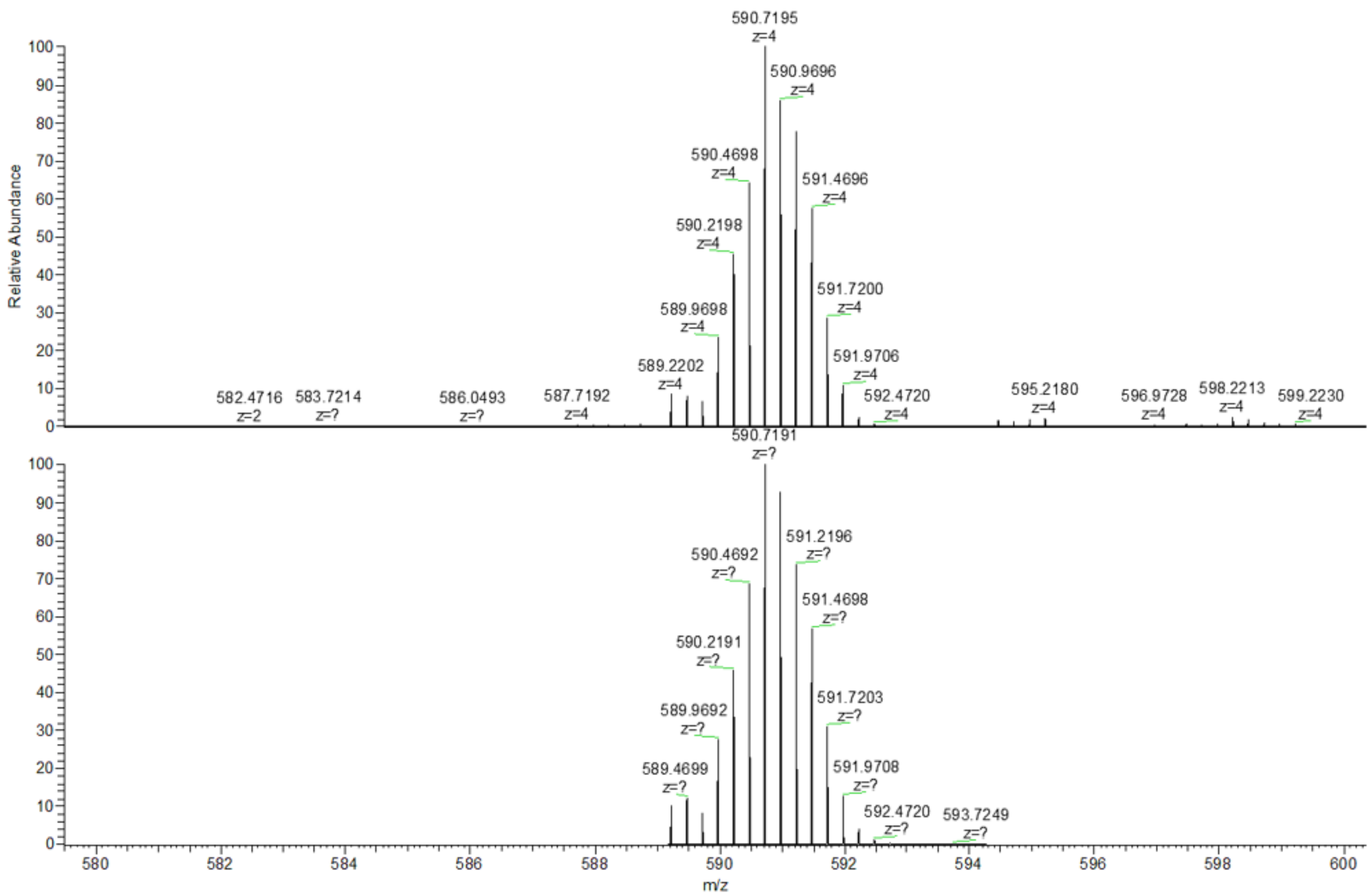

Fig. S3. *ESI-MS of $[CB7–Rubpy – C_8(mim)_2]^{4+}$ adduct, measured (top) and simulated (bottom).*

3. Supporting data and figures

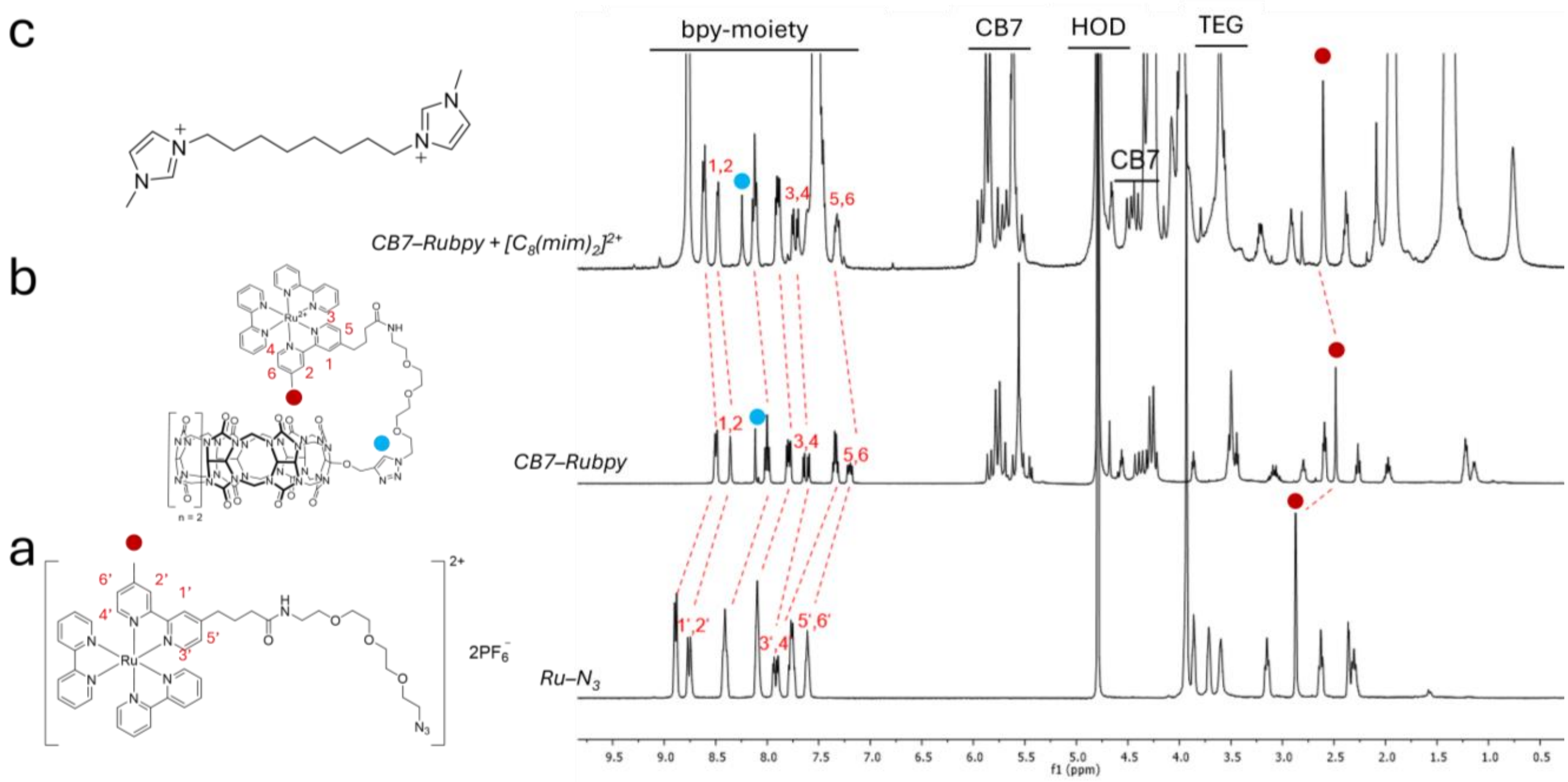

Fig. S4. *Overlay of* [1]*H–NMR spectra (400 MHz, in D_2O) of a) the azide-functionalized complex of the Ru(II) precursor, i.e., Ru–N_3, 2 mM, b) CB7–Rubpy 2 mM alone, c) CB7–Rubpy 2 mM host–guest association with 10 equivalents of $[C_8(mim)_2]^{2+}$.*

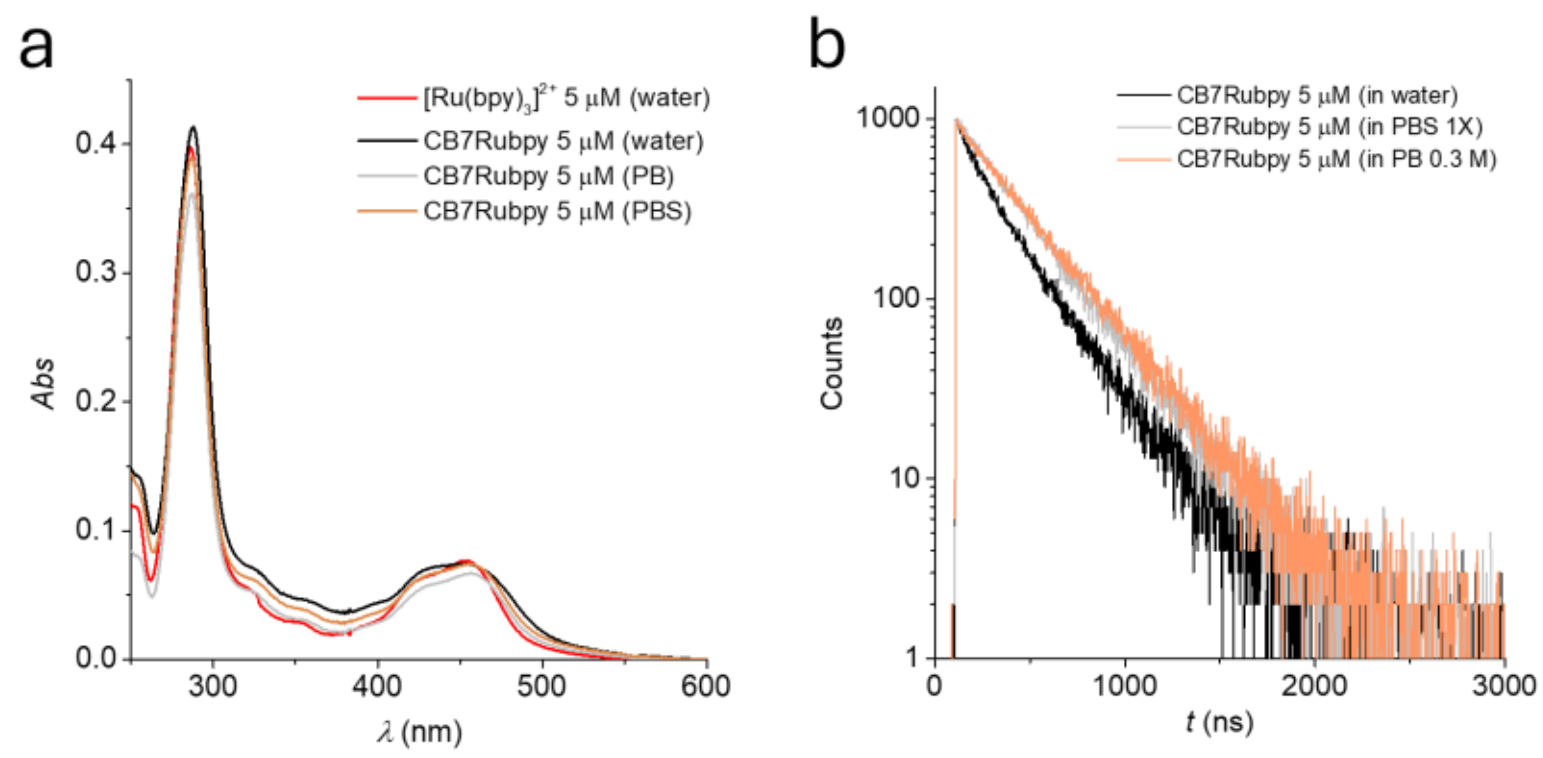

c

Sample	τ ± St. Dev. (ns)	B (Rel. %)	χ^2
CB7Rubpy 5 µM (in water)	91.51 ± 8.33<br>275.47 ± 3.37	282.7 (12.1)<br>682.5 (87.9)	0.99
CB7Rubpy 5 µM (in PBS 1X)	304.12 ± 1.01	985.8 (100)	1
CB7Rubpy 5 µM (in PB 0.3 M)	327.04 ± 1.12	927.8 (100)	1

Fig. S5. *Photophysical characterization of CB7–Rubpy in different solvents, in comparison to a standard $[Ru(bpy)_3]^{2+}$. a) Absorption spectra of $[Ru(bpy)_3]^{2+}$ 5 µM (in water, red), and CB7–Rubpy 5 µM (in water, black; in PB 0.3 M pH 6.8, grey; in PBS 1X pH 7.4, orange). b) Emissive lifetime decays of CB7–Rubpy 5 µM in water (black), PBS 1X (grey), and PB 0.3 M (orange); λ_{exc} = 475 nm, λ_{em} = 620 nm, bandwidth = 3 nm. c) Fitted lifetime values of the investigated samples.*

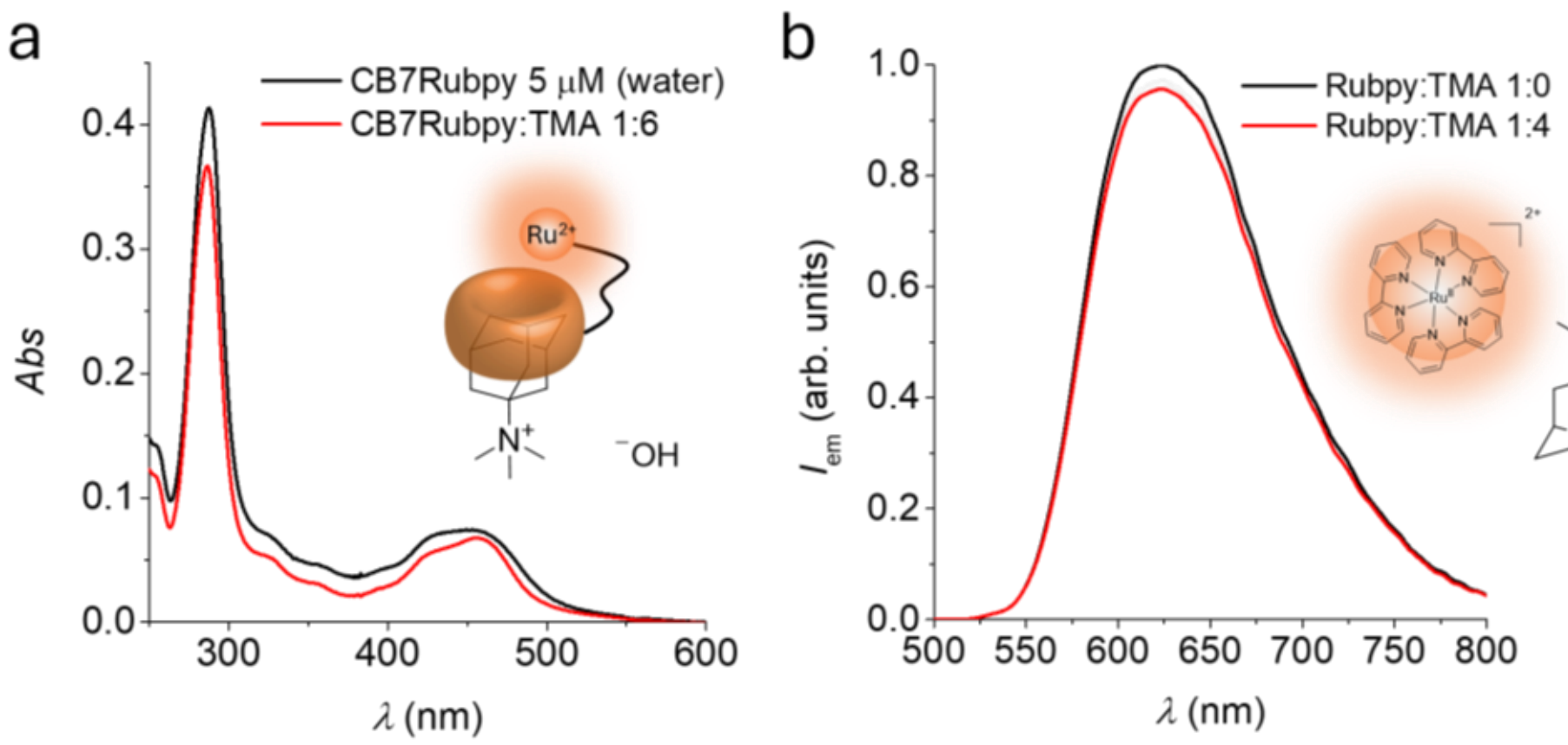

Fig. S6. *a) Absorption spectra of CB7–Rubpy 5 μM upon addition of increasing amounts (1–6 equivalents) of TMA in water (related to the photoluminescent response reported in **Fig. 2b**). b) Emission spectra of a solution of $[Ru(bpy)_3]^{2+}$ 5 μM upon addition of increasing amounts (1–4 eq.) of TMA in water. For all the spectra, λ_{exc} = 450 nm, excitation and emission slits = 10 nm, emission cut-off = 475 nm, normalised on the maximum emission intensity of the highest–response sample.*

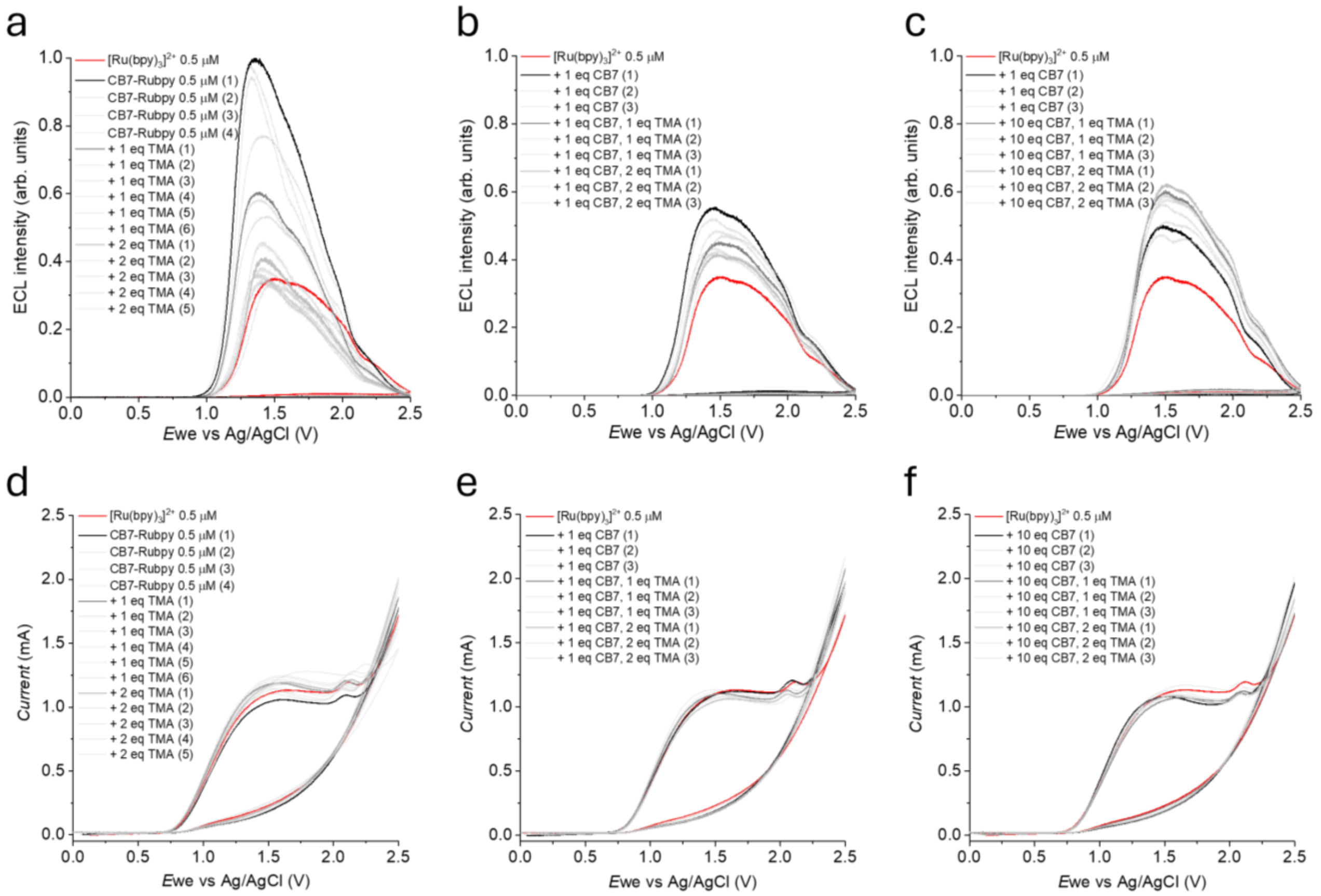

Fig. S7. *CV–ECL measurements performed in a 0.3 M PB solution at pH 6.8, with TPrA 180 mM (relative to the ECL intensities reported in **Fig. 3**). ECL intensity vs applied potential is reported for a) CB7–Rubpy 0.5 μM, either alone or in the presence of 1–2 eq. of TMA; b) $[Ru(bpy)_3]^{2+}$ 0.5 μM in the presence of 1 equivalent of CB7, upon addition of 1–2 equivalents of TMA; c) $[Ru(bpy)_3]^{2+}$ 0.5 μM in the presence of 10 equivalents of CB7, upon addition of 1–2 eq. of TMA. The working electrode potential was scanned at 100 mV/s, and emission acquired every 100 ms (control voltage 750 mV, photosensor module positioned on the short side of the cell). Amplification level at 00.0 μA. Corresponding CV plots are reported as well for d) CB7–Rubpy 0.5 μM, either alone or in the presence of 1–2 eq. of TMA; e) $[Ru(bpy)_3]^{2+}$ 0.5 μM in the presence of 1 equivalent of CB7, upon addition of 1–2 equivalents of TMA; f) $[Ru(bpy)_3]^{2+}$ 0.5 μM in the presence of 10 equivalents of CB7, upon addition of 1–2 eq. of TMA.*

Table S1. *Relative charge-normalized ECL values of CB7–Rubpy and* $[Ru(bpy)_3]^{2+}$*, obtained by integrating the ECL emission and the faradaic current over the same potential window (0.75–2.5 V) of the CV–ECL experiments reported in* ***Fig. 3a*** *and* ***Fig. S7a*** *and* ***S7d*** *(performed in a 0.3 M PB solution at pH 6.8, with TPrA 180 mM and and* $[Ru(bpy)_3]^{2+}$*/CB7–Rubpy 0.5 µM, scan rate 100 mV/s). Since the potential is swept linearly and the scan rate is the same for all measurements, the ratio* $\frac{\int ECL{\cdot}dE}{\int i{\cdot}dE}$ *is proportional to the light emitted per unit charge. The limits of the integration window correspond to the onset of the faradaic current and to the return of the ECL emission to the baseline; the same window is applied to the emission and to the current, and to all the compared systems.*

**Sample**	$\int i \cdot dE$ **(arb.units)**	$\int ECL \cdot dE$ **(arb.units)**	**Charge-normalized ECL response (arb. units)**
$[Ru(bpy)_3]^{2+}$ 0.5 µM	1.69	0.28	0.17
CB7–Rubpy 0.5 µM (1)	1.59	0.54	0.34
CB7–Rubpy 0.5 µM (2)	1.75	0.58	0.33
CB7–Rubpy 0.5 µM (3)	1.71	0.66	0.39
CB7–Rubpy 0.5 µM (4)	1.77	0.53	0.30

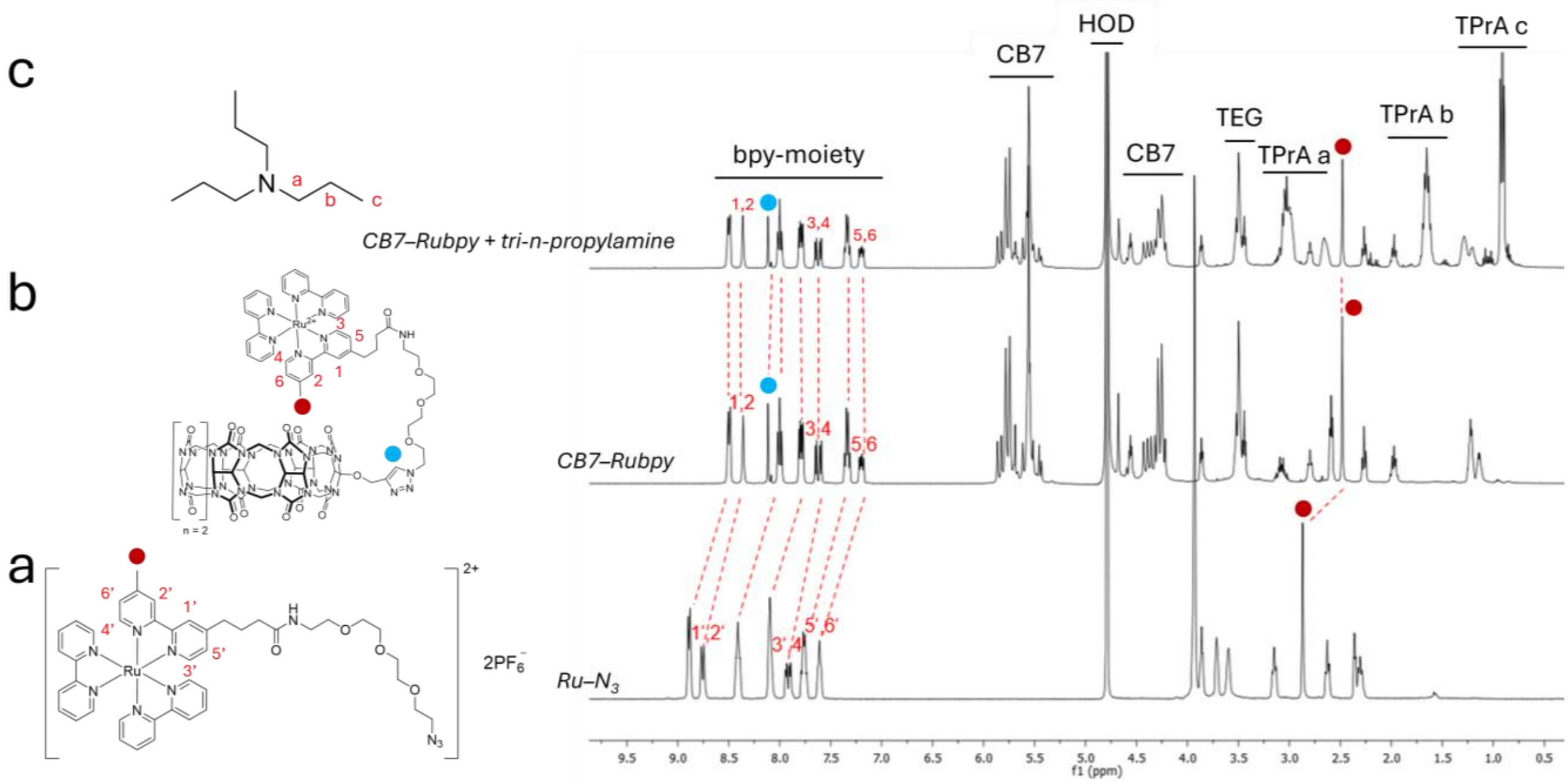

Fig. S8. *Overlay of* ^{1}*H–NMR spectra (400 MHz, in* D_2O*) of a) the azide-functionalized complex of the Ru(II) precursor, i.e., Ru–*N_3*, 2 mM, b) CB7–Rubpy 2 mM alone, c) CB7–Rubpy 2 mM in the presence of TPrA 20 mM. Global shifting of all signals confirms a slight (not full) unfolding of CB7–Rubpy (considering that the ratio TPrA:CB7–Rubpy here is lower than the one used in a typical ECL experiment).*

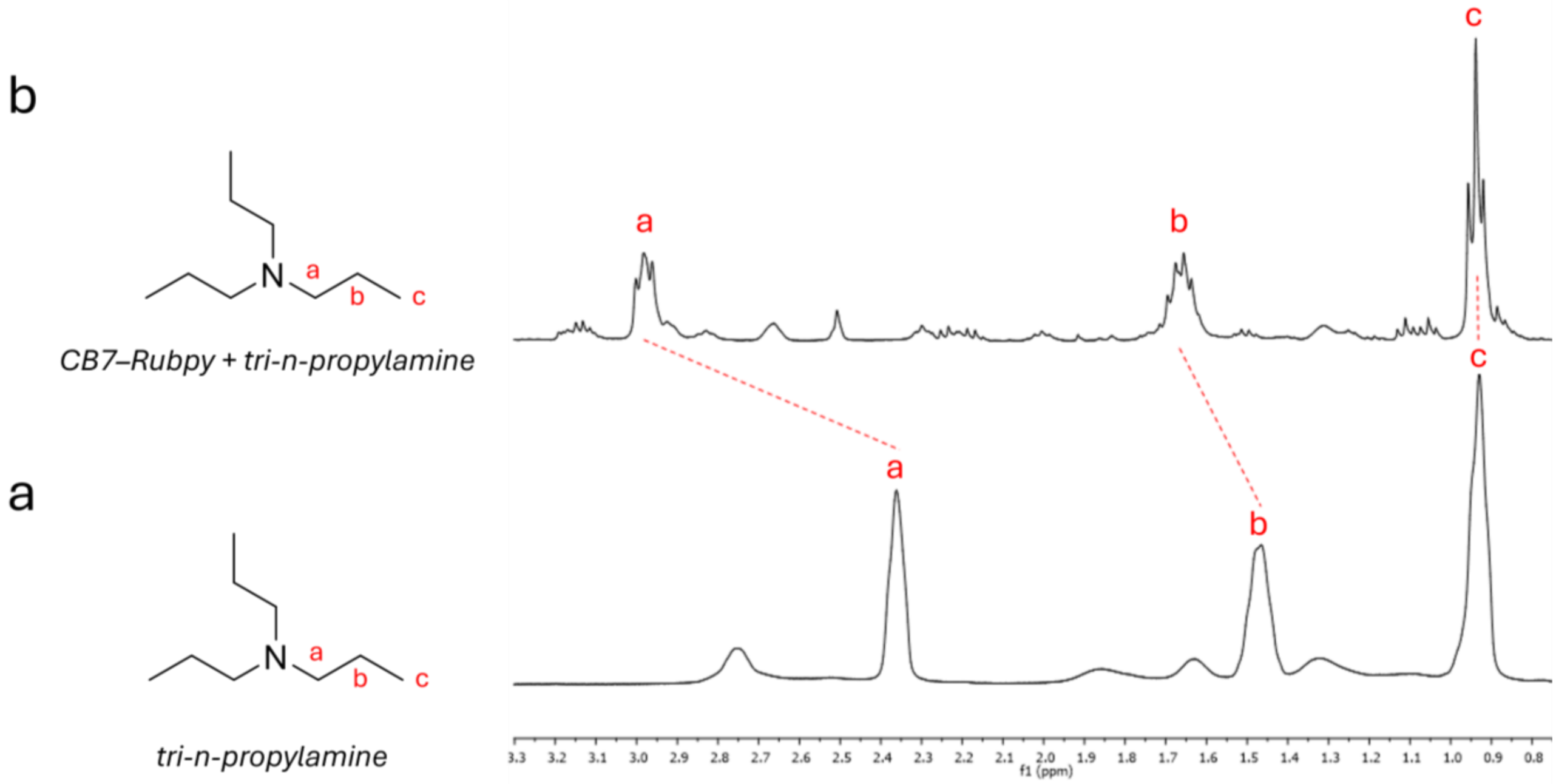

Fig. S9. *Overlay of ^{1}H–NMR spectra (400 MHz, in D_2O) of tri-n-propylamine either a) alone, 2 mM, or b) in the presence of CB7–Rubpy (CB7–Rubpy 2 mM, TPrA 30 mM). To be noted that the TPrA:CB7–Rubpy ratio was not as high as the one typically used in a ECL experiment, when TPrA is commonly employed in a much larger excess.*

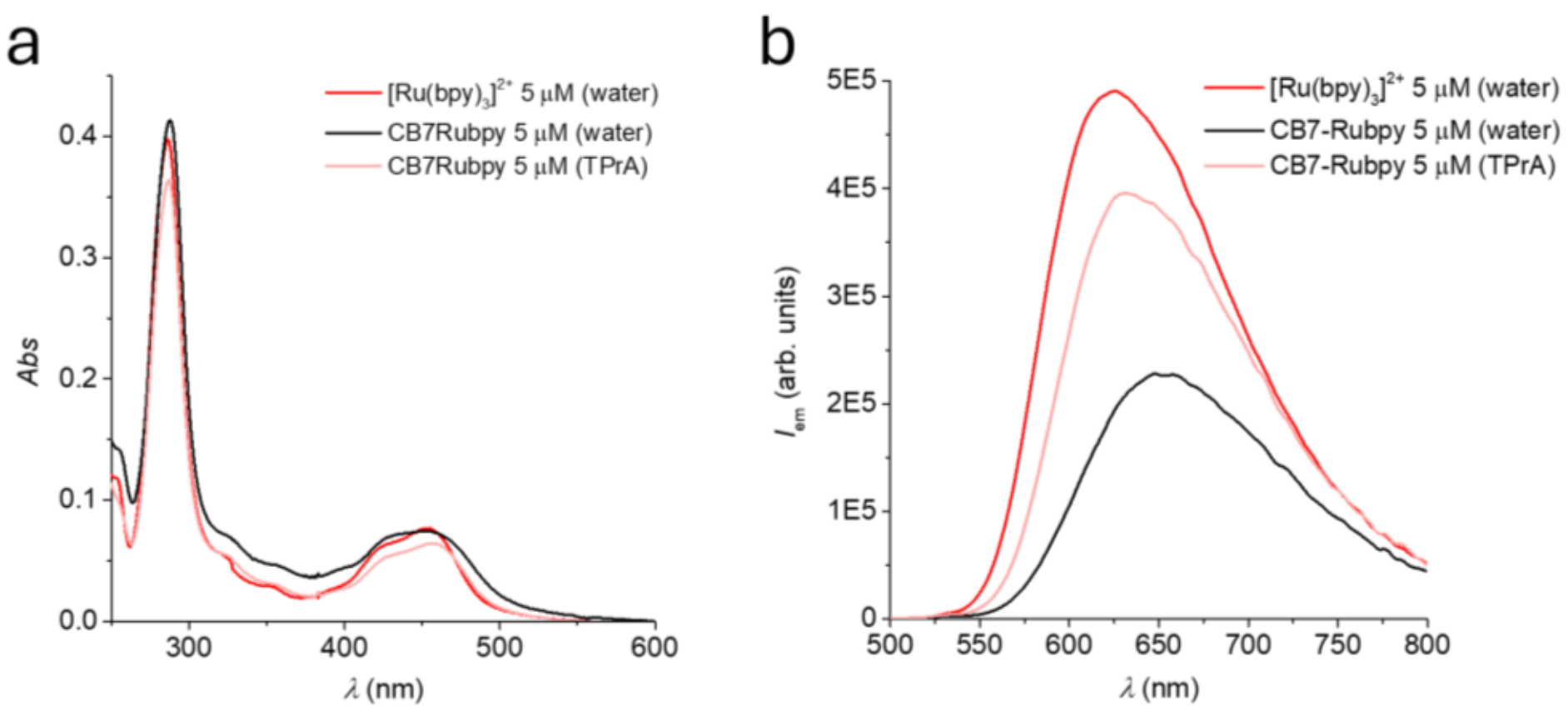

Fig. S10. *Photophysical characterization of CB7–Rubpy in ECL experimental conditions, in comparison to a standard $[Ru(bpy)_3]^{2+}$. a) Absorption spectra of $[Ru(bpy)_3]^{2+}$ 5 μM (in water, red), and CB7–Rubpy 5 μM (in water, black; in the presence of TPrA 180 mM in PB 0.3 M pH 6.8, pink). b) Emission spectra of $[Ru(bpy)_3]^{2+}$ 5 μM (in water, red), and CB7–Rubpy 5 μM (in water, black; in the presence of TPrA 180 mM in PB 0.3 M pH 6.8, pink). For all the spectra, λ_{exc} = 450 nm, excitation and emission slits = 10 nm, emission cut-off = 475 nm.*

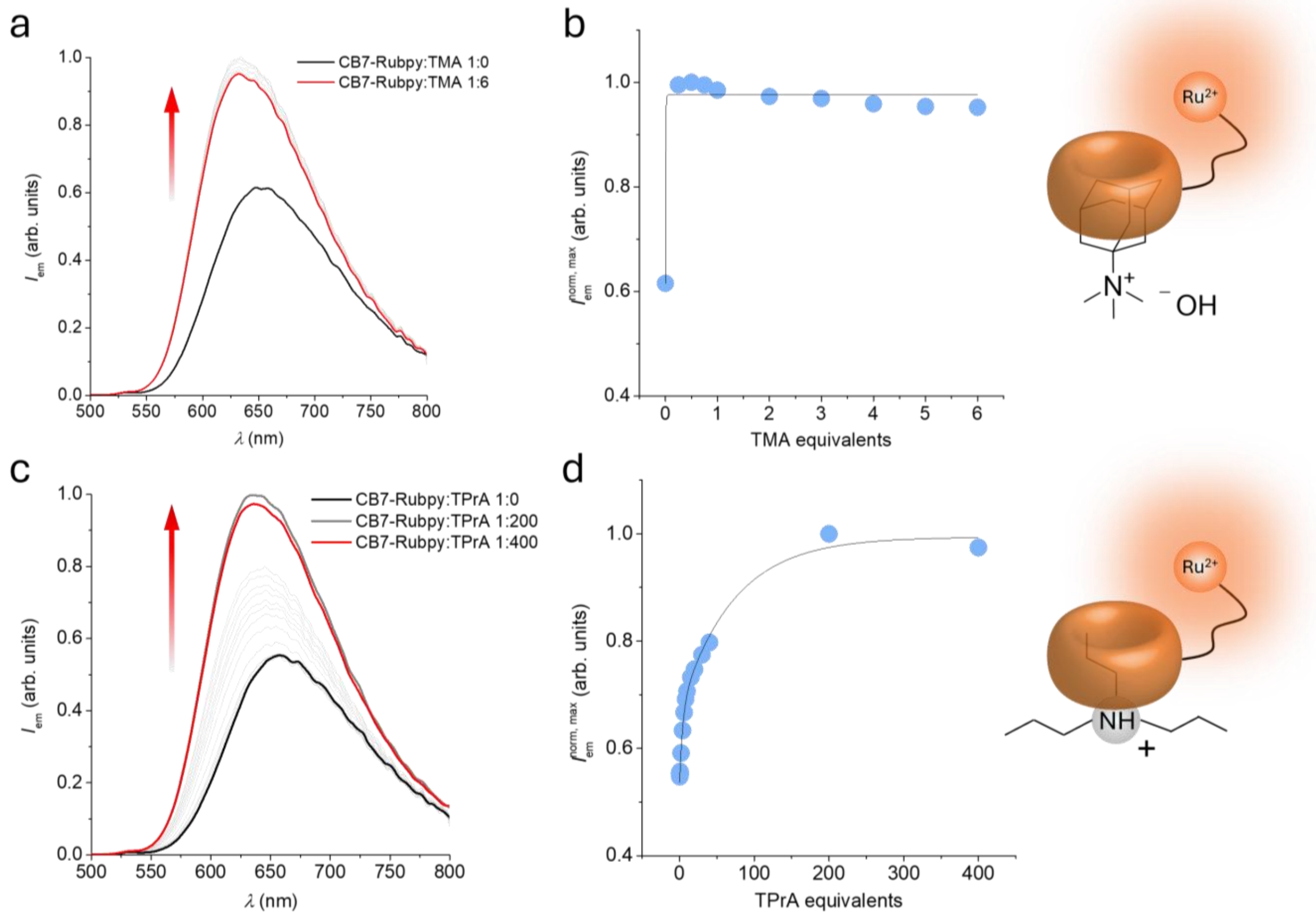

Fig. S11. *Comparison of the photoluminescent response upon host–guest association between a solution of CB7–Rubpy 5 μM (in water) and either a) increasing amount (1–6 eq.) of TMA (with b) reporting the corresponding maximum emission intensity vs guest added equivalents fitting), or c) increasing amount (1–400 eq.) of TPrA (with d) reporting the corresponding maximum emission intensity vs guest added equivalents fitting). For all the spectra, λ_{exc} = 450 nm, excitation and emission slits = 10 nm, emission cut-off = 475 nm, normalised on the maximum emission intensity of the highest–response sample.*

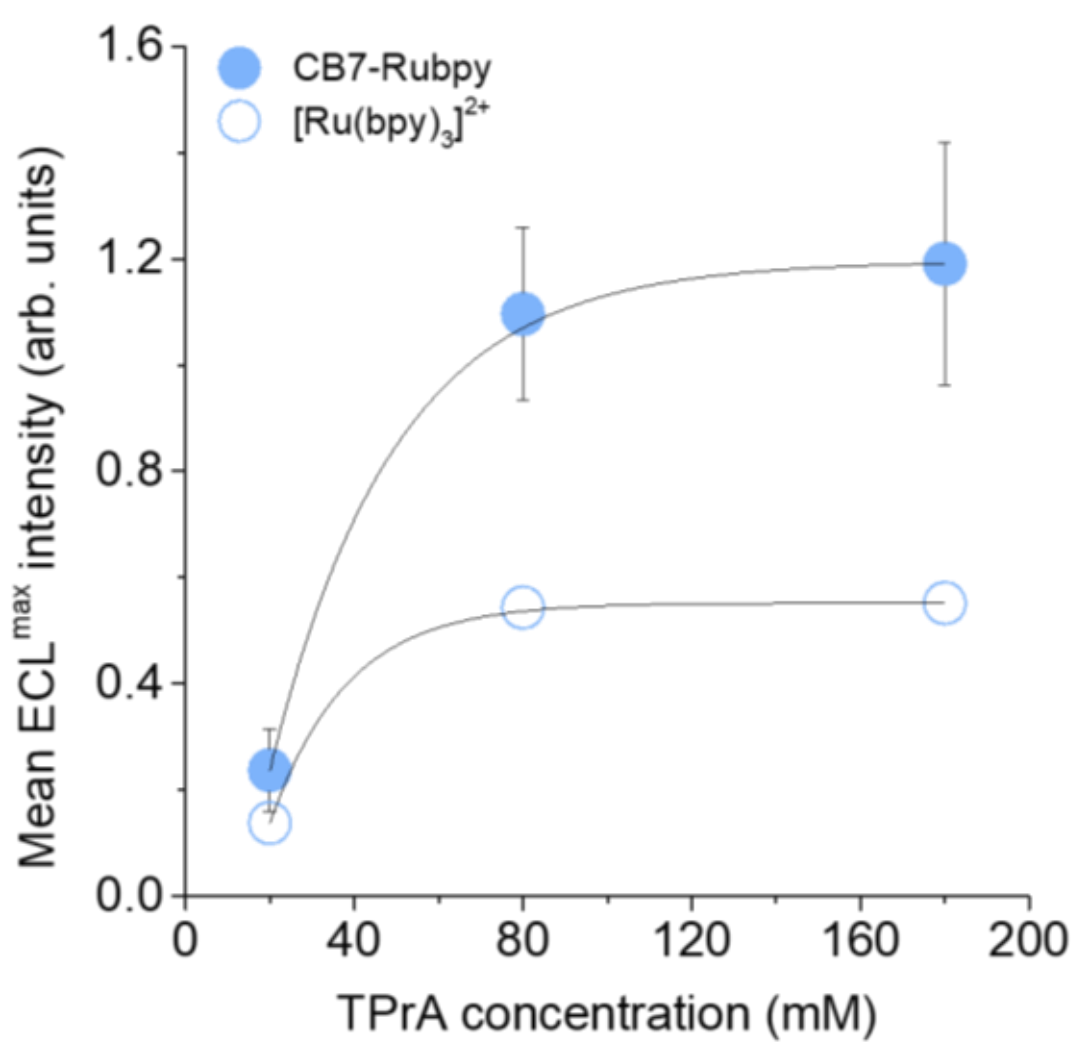

Fig. S12. *Mean value of the maximum ECL intensity obtained upon increasing TPrA concentration (20–180 mM) either in the presence of $[Ru(bpy)_3]^{2+}$ 0.5 μM as luminophore (empty dots) or in the presence of CB7–Rubpy 0.5 μM as luminophore (filled dots). CV–ECL measurements performed in a 0.3 M PB solution at pH 6.8 (SD calculated for n ≥ 3). The working electrode potential was scanned at 100 mV/s, and emission acquired every 100 ms (control voltage 750 mV, photosensor module positioned on the short side of the cell). Amplification level at 00.0 μA.*

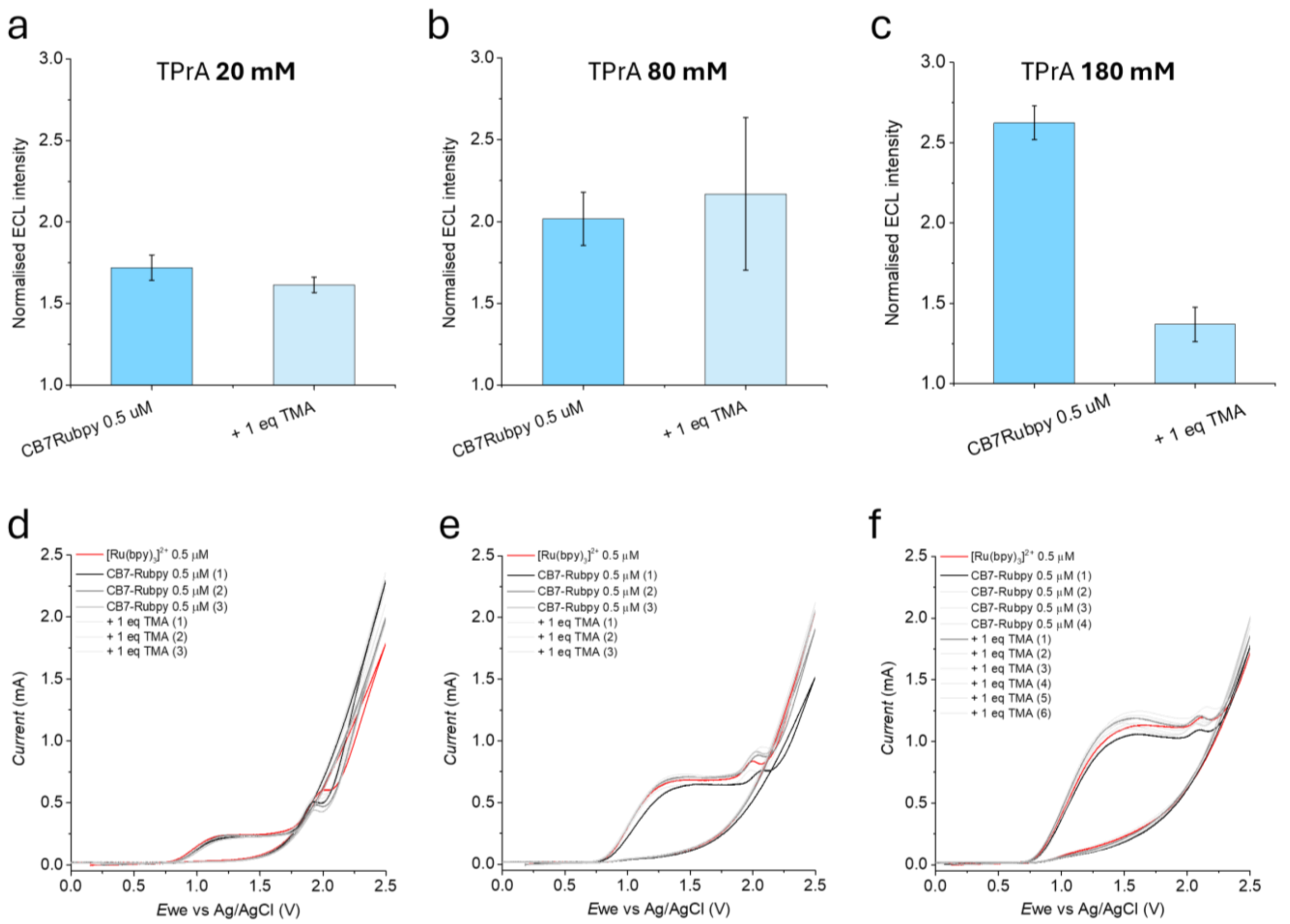

Fig. S13. *CV–ECL measurements performed on $[Ru(bpy)_3]^{2+}$ 0.5 µM, and CB7–Rubpy 0.5 µM, either alone or in the presence of 1 equivalent of TMA, in a 0.3 M PB solution at pH 6.8, upon increasing TPrA concentration. The working electrode potential was scanned at 100 mV/s, and emission acquired every 100 ms (control voltage 750 mV, photosensor module positioned on the short side of the cell). Amplification level at 00.0 µA. The resulting ECL signal intensity is normalised on the $[Ru(bpy)_3]^{2+}$ 0.5 µM ECL signal (which thus corresponds to 1), and reported in the presence of: a) TPrA 20 mM; b) TPrA 80 mM; c) TPrA 180 mM (SD calculated for n ≥ 3). Corresponding CV plots are reported as well, corresponding to the presence of d) TPrA 20 mM; e) TPrA 80 mM; f) TPrA 180 mM.*

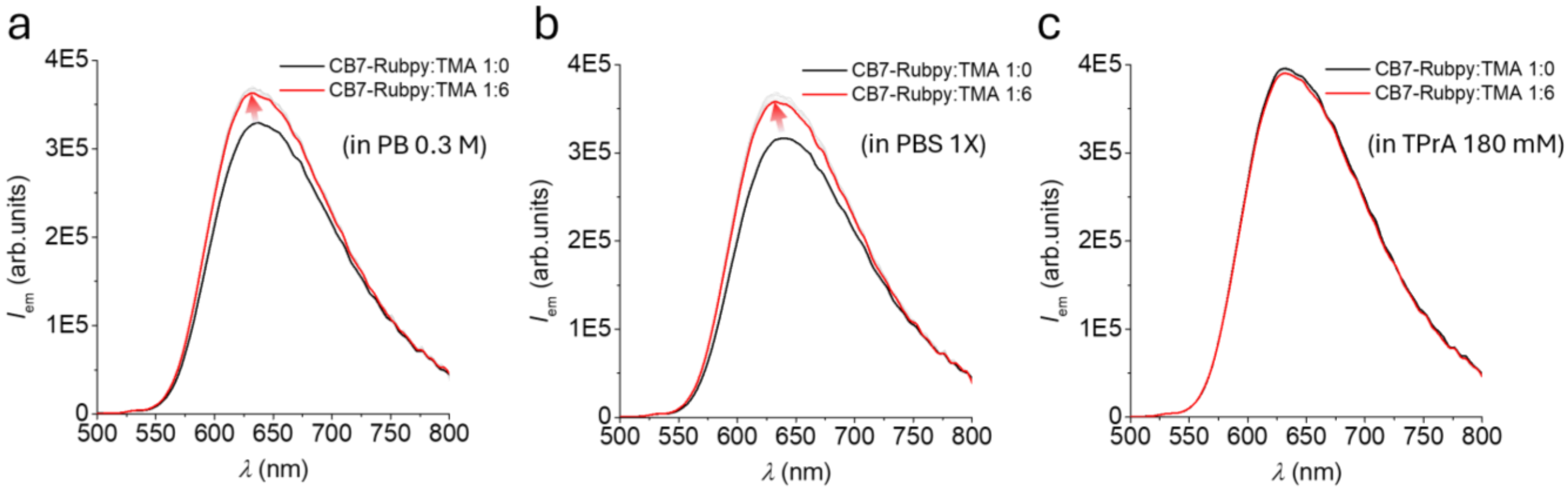

Fig. S14. *Emission spectra of a solution of CB7–Rubpy 5 µM upon addition of increasing amounts (1–6 equivalents) of TMA in a) PB 0.3 M pH 6.8, b) PBS 1X pH 7.4, c) TPrA 180 mM in PB 0.3 M pH 6.8. For all the spectra, λ_{exc} = 450 nm, excitation and emission slits = 10 nm, emission cut-off = 475 nm.*

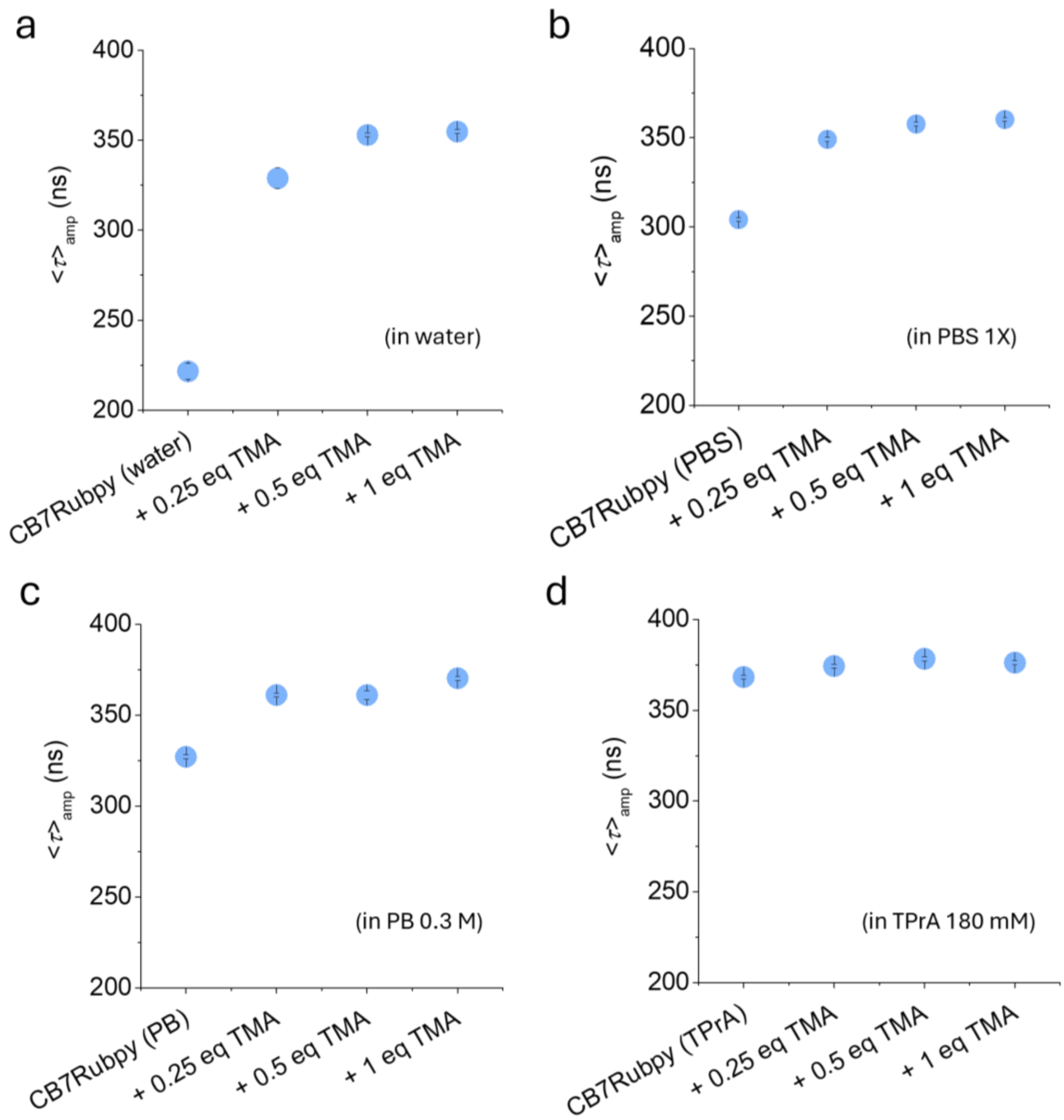

Fig. S15. *Mean values of <τ>$_{amp}$, i.e., the average lifetime weighted on amplitude (SD calculated for n ≥ 3), of a solution of CB7–Rubpy 5 µM upon addition of increasing amounts (0.25–1 eq.) of TMA either in a) water, b) PBS 1X, c) PB 0.3 M, d) TPrA 180 mM in PB 0.3 M pH 6.8.*

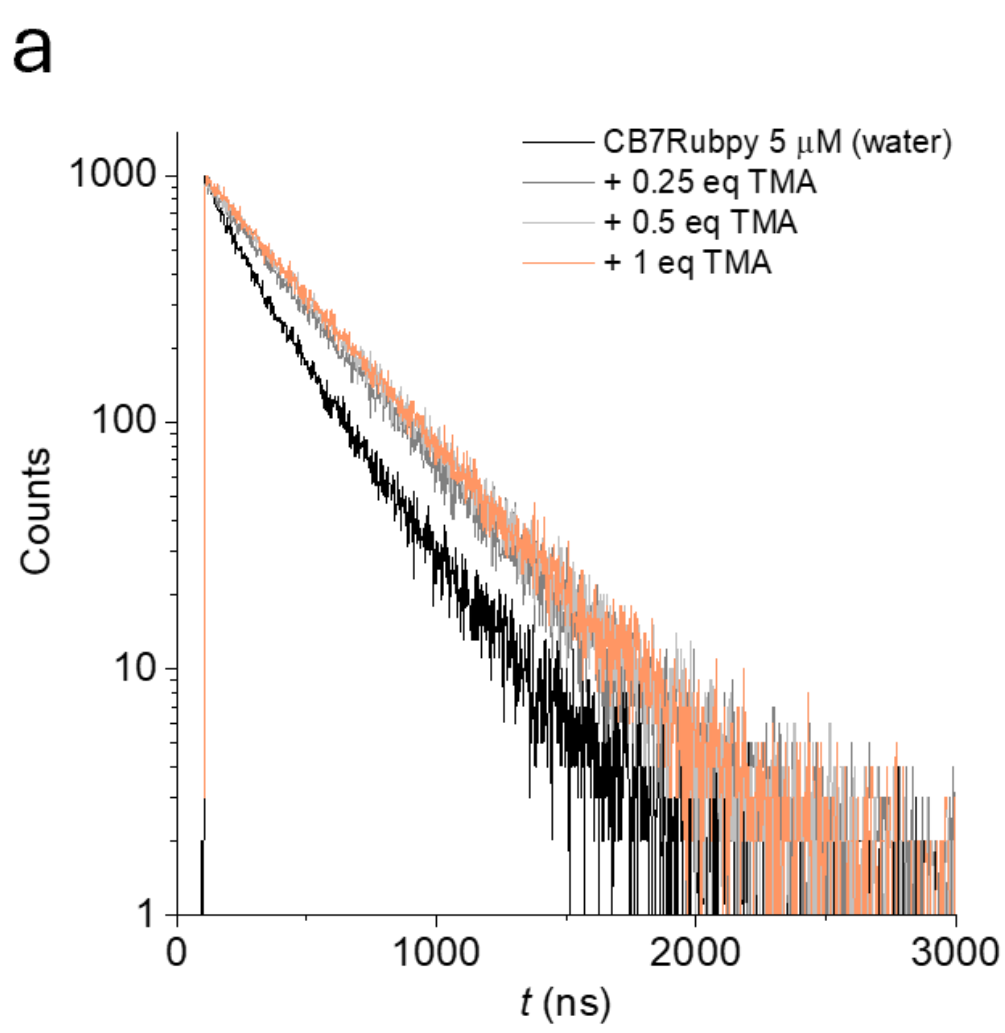

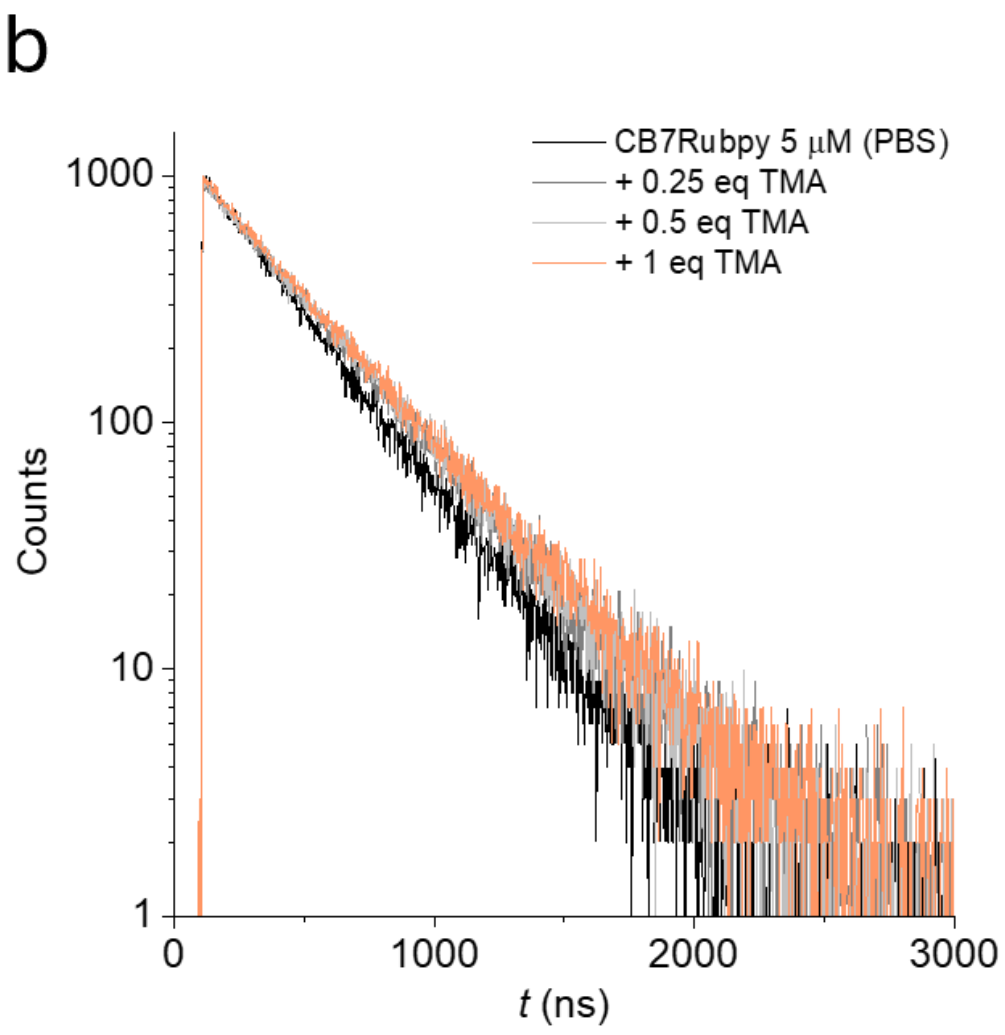

Sample	τ ± St. Dev. (ns)	B (Rel. %)	χ^2
CB7Rubpy 5 μM (water)	91.51 ± 8.33<br>275.47 ± 3.37	282.7 (12.1)<br>682.5 (87.9)	0.99
+ 0.25 eq TMA	116.79 ± 46.5<br>344.51 ± 2.86	62.28 (2.4)<br>844.36 (97.6)	0.99
+ 0.5 eq TMA	352.87 ± 1.09	934.93 (100)	1
+ 1 eq TMA	354.75 ± 1.19	959.44 (100)	0.99

Sample	τ ± St. Dev. (ns)	χ^2
CB7Rubpy 5 μM (PBS)	304.12 ± 1.01	1
+ 0.25 eq TMA	349.07 ± 1.11	1
+ 0.5 eq TMA	357.67 ± 1.08	1
+ 1 eq TMA	360.21 ± 1.11	0.99

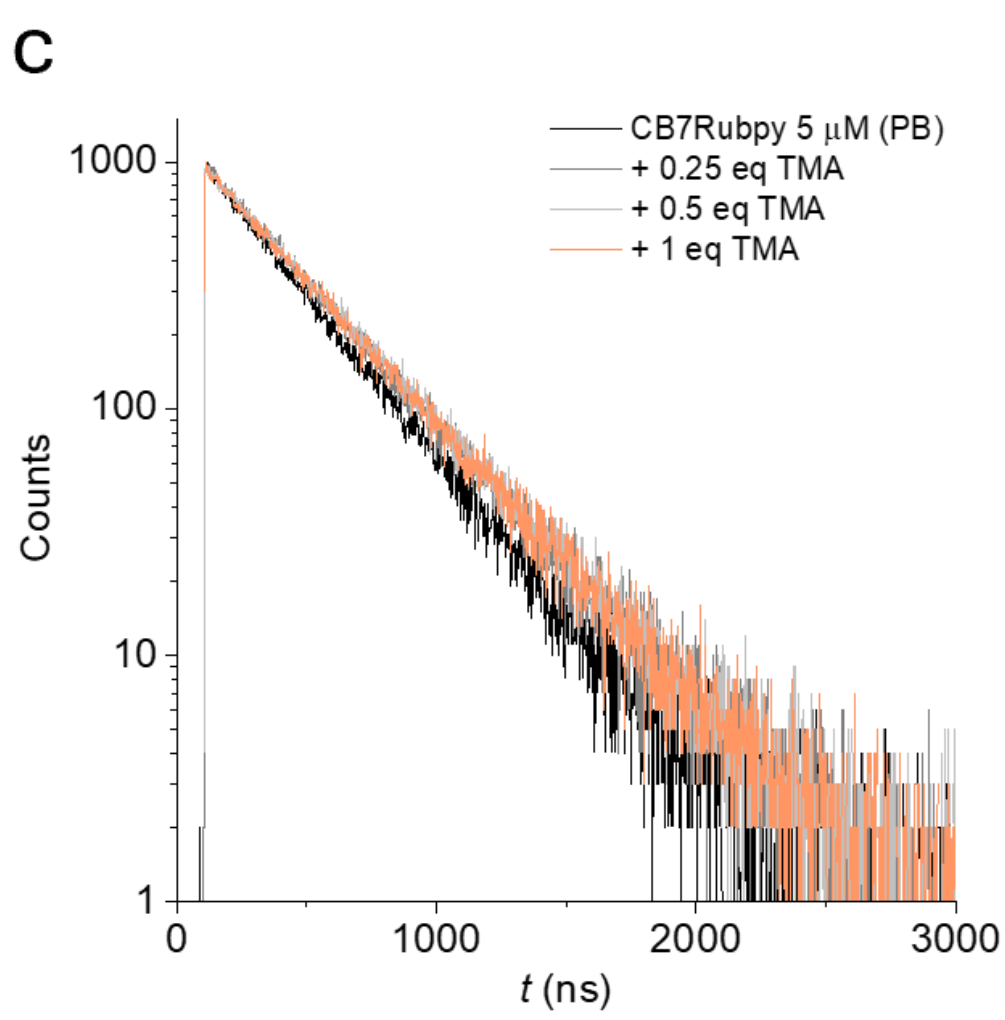

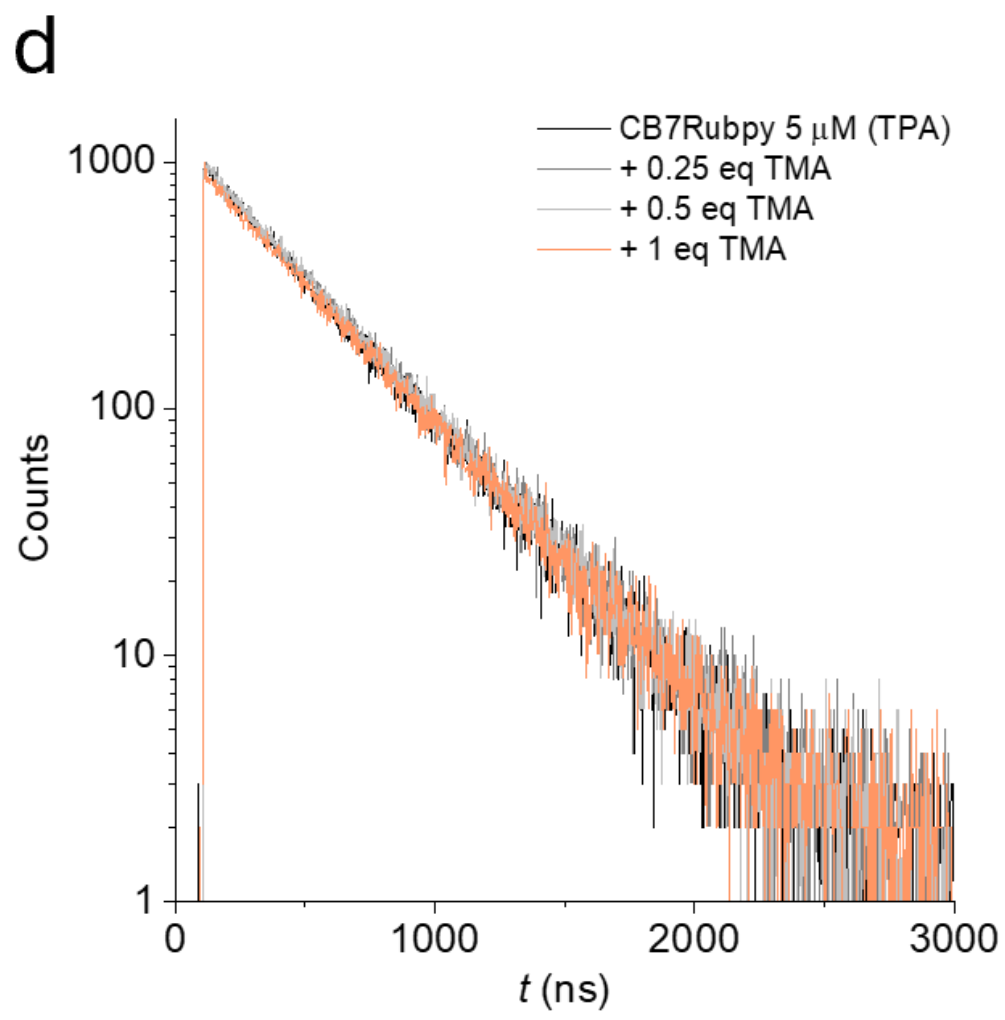

Sample	τ ± St. Dev. (ns)	χ^2
CB7Rubpy 5 μM (PB)	327.04 ± 1.12	1
+ 0.25 eq TMA	361.01 ± 1.11	0.99
+ 0.5 eq TMA	361 ± 0.00	0.99
+ 1 eq TMA	370.23 ± 1.14	1

Sample	τ ± St. Dev. (ns)	χ^2
CB7Rubpy 5 μM (TPA)	368.33 ± 1.11	1
+ 0.25 eq TMA	374.38 ± 1.13	1
+ 0.5 eq TMA	378.38 ± 1.19	0.99
+ 1 eq TMA	376.25 ± 1.17	1

Fig. S16. *Emissive lifetime decays of CB7–Rubpy 5 μM upon addition of increasing amounts (0.25–1 eq.) of TMA in a) water, b) PBS 1X, c) PB 0.3 M, d) TPrA 180 mM in PB 0.3 M pH 6.8. The related fitted lifetime values are reported below each investigated sample. For all the samples,* λ_{exc} *= 475 nm,* λ_{em} *= 620 nm, bandwidth = 3 nm.*

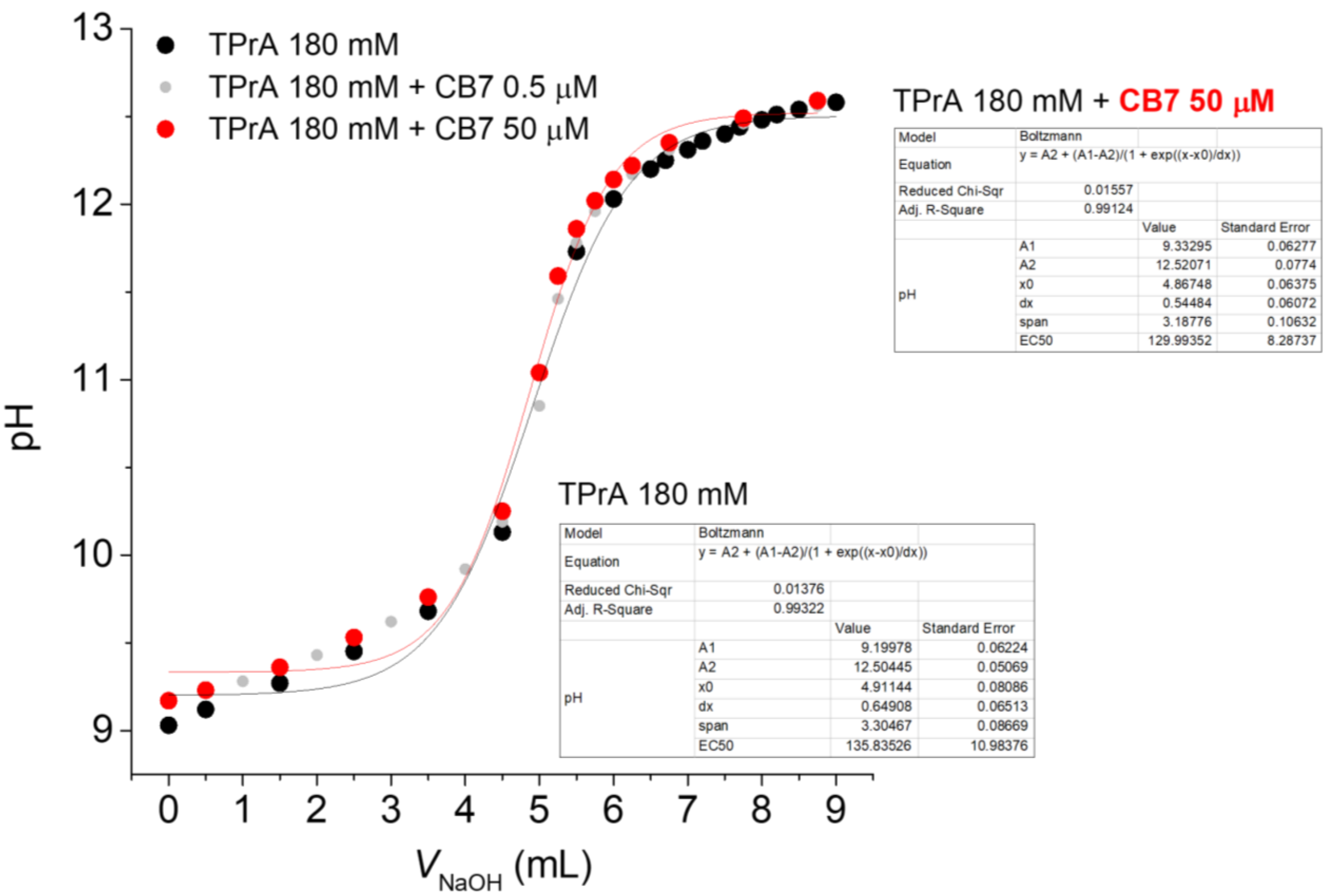

Fig. S17. *Acid–base titration performed on an aqueous solution (PB 0.3 M) of TPrA 180 mM fully protonated ($TPrAH^+$, addition of 1 eq. of HCl), either alone or in the presence of CB7 0.5 or 50 µM, upon increasing addition of NaOH 0.5 M (V_{NaOH}=volume of added NaOH, in mL). The obtained curves have been fitted with a single Boltzmann model, and the corresponding fitting parameters have been reported.*

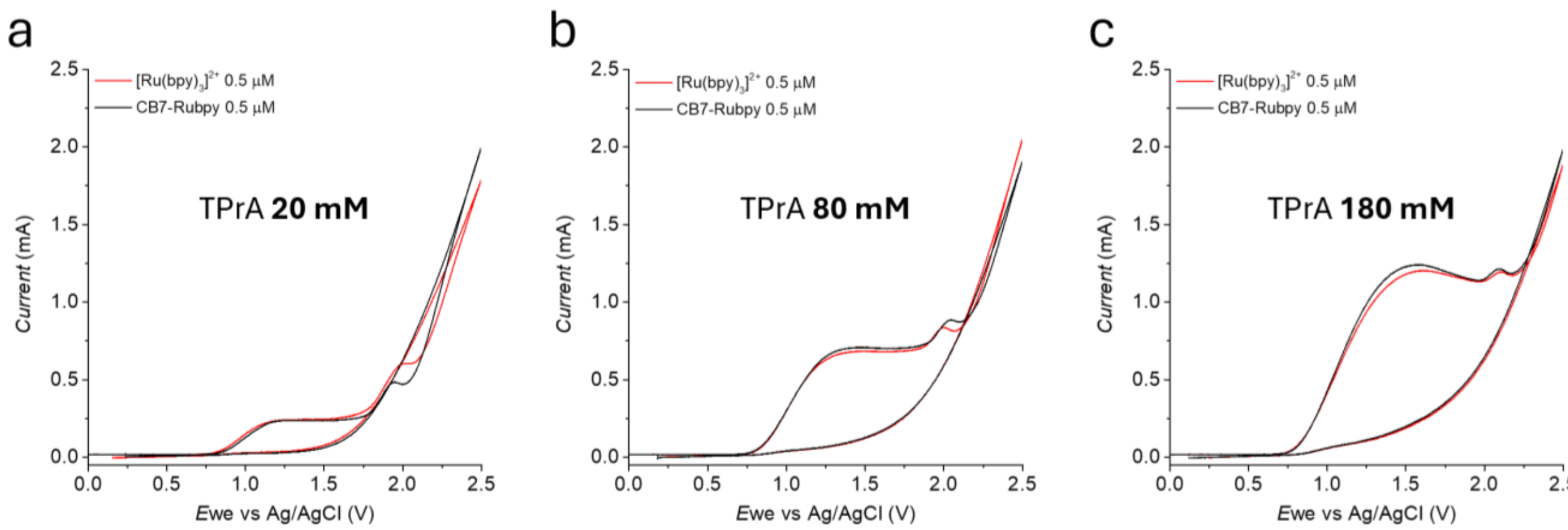

Fig. S18. *Comparison of CV–ECL measurements performed either on $[Ru(bpy)_3]^{2+}$ 0.5 µM (red) or CB7–Rubpy 0.5 µM (black), in a 0.3 M PB solution at pH 6.8, upon increasing TPrA concentration: a) TPrA 20 mM; b) TPrA 80 mM; c) TPrA 180 mM. The working electrode potential was scanned at 100 mV/s.*

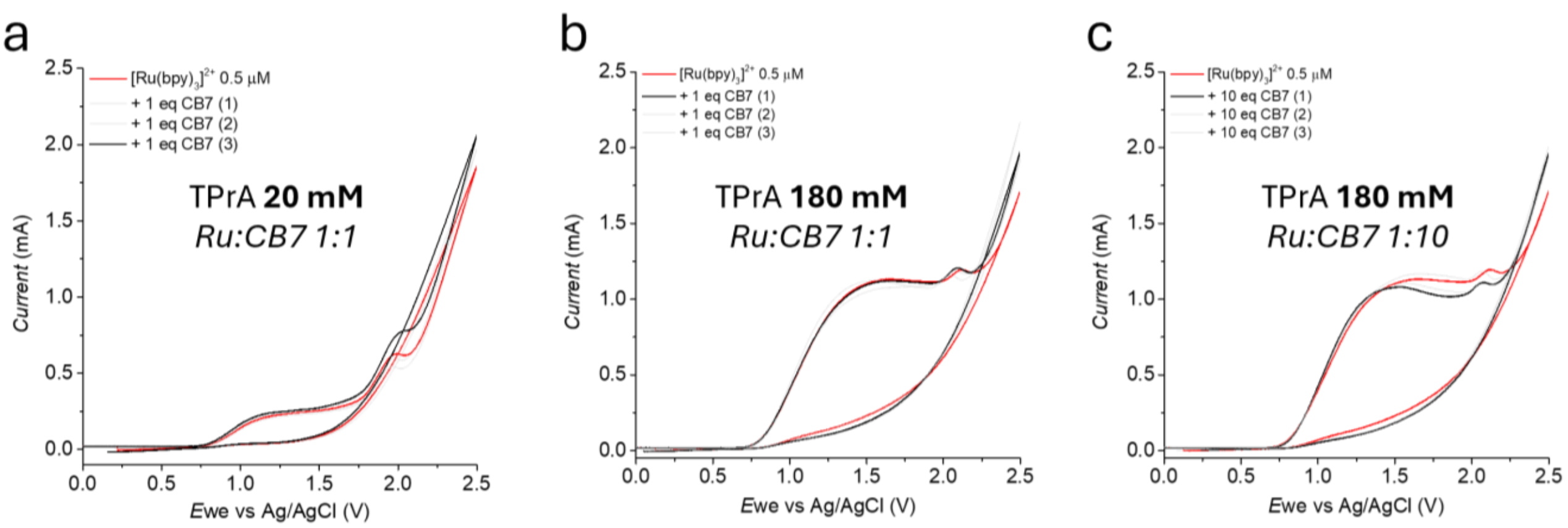

Fig. S19. *Comparison of CV–ECL measurements performed on [Ru(bpy)$_3$]$^{2+}$ 0.5 μM either alone (red) or upon addition of CB7 (black, 1 or 10 eq.), in a 0.3 M PB solution at pH 6.8, upon increasing TPrA concentration: a) TPrA 20 mM, 1 eq. of CB7; b) TPrA 180 mM, 1 eq. of CB7; c) TPrA 180 mM, 10 eq. of CB7. The working electrode potential was scanned at 100 mV/s.*

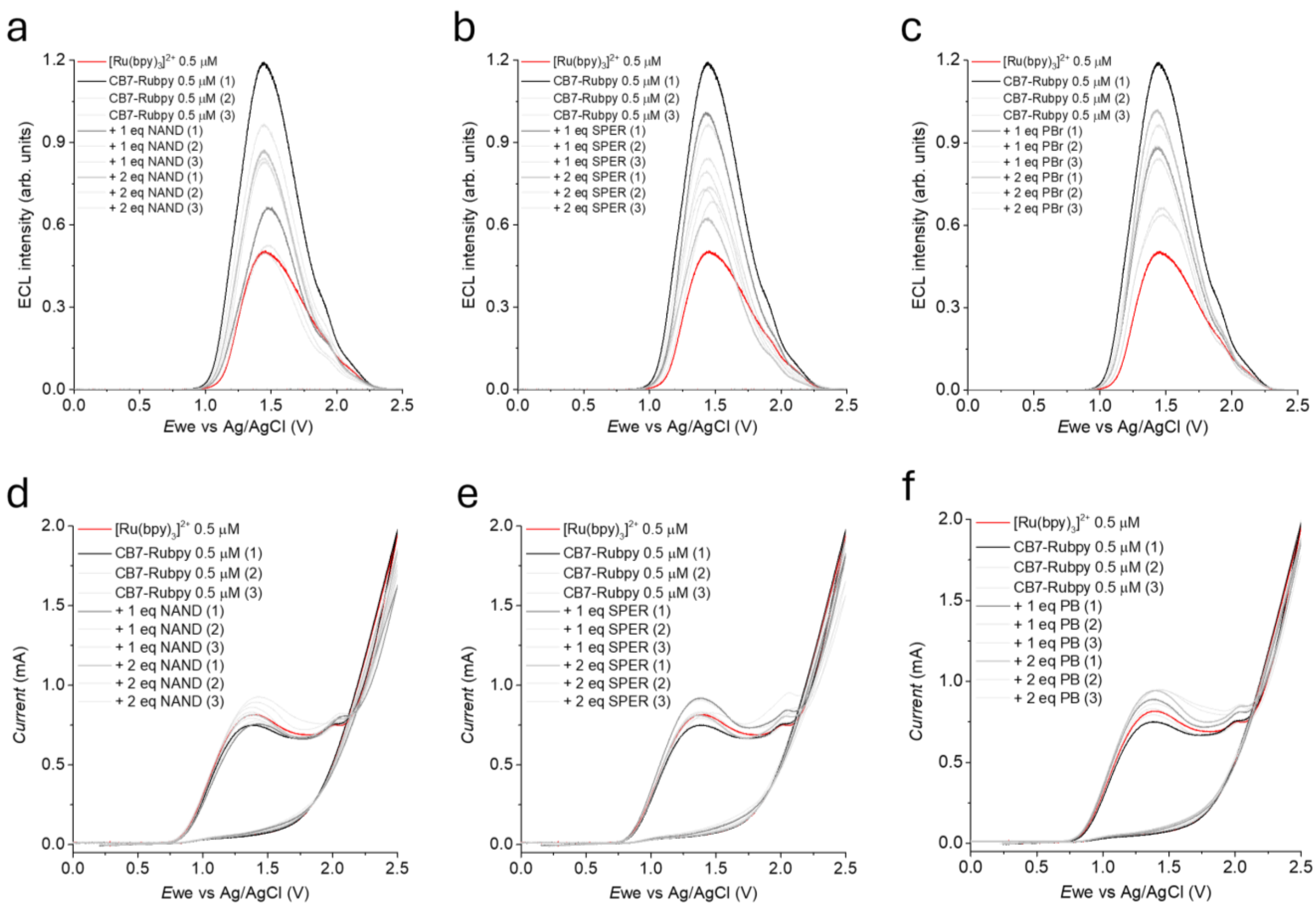

Fig. S20. *CV–ECL measurements performed in a 0.3 M PB solution at pH 6.8, with TPrA 180 mM (relative to the ECL intensities reported in **Fig. 6**). ECL intensity vs applied potential is reported for a) CB7–Rubpy 0.5 μM, either alone or in the presence of 1–2 eq. of nandrolone; b) CB7–Rubpy 0.5 μM, either alone or in the presence of 1–2 eq. of spermidine trihydrochloride; c) CB7–Rubpy 0.5 μM, either alone or in the presence of 1–2 eq. of pancuronium bromide. The working electrode potential was scanned at 100 mV/s, and emission acquired every 100 ms (control voltage 750 mV, photosensor module positioned on the short side of the cell). Amplification level at 00.0 μA. Corresponding CV plots are reported as well for d) CB7–Rubpy 0.5 μM, either alone or in the presence of 1–2 eq. of nandrolone; e) CB7–Rubpy 0.5 μM, either alone or in the presence of 1–2 eq. of spermidine trihydrochloride; f) CB7–Rubpy 0.5 μM, either alone or in the presence of 1–2 eq. of pancuronium bromide.*

a

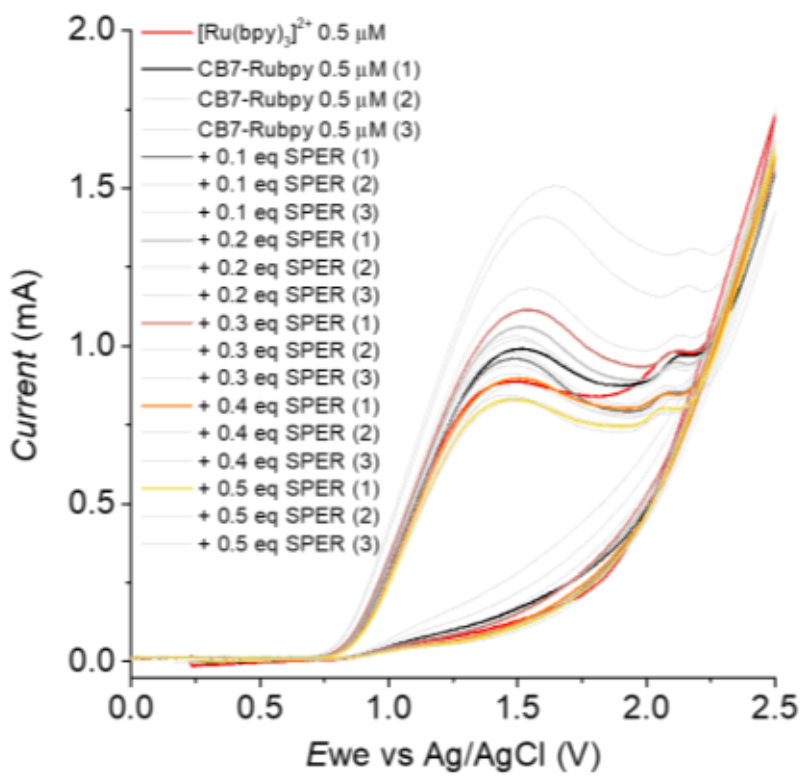

b

Equation	y = a + b*x		
Weight	Instrumental		
Residual Sum of Squares	4.15875		
Pearson's r	-0.97948		
Adj. R-Square	0.94584		
		Value	Standard Error
ECL mean intensity	Intercept	2.29706	0.14668
ECL mean intensity	Slope	-6.43794	0.76485

Fig. S21. *CV–ECL measurements performed in a 0.3 M PB solution at pH 6.8, with TPrA 180 mM (relative to the ECL calibration curve reported in* ***Fig. 7****). The working electrode potential was scanned at 100 mV/s, and emission acquired every 100 ms (control voltage 750 mV, photosensor module positioned on the short side of the cell). Amplification level at 00.0 µA. a) CV plots for CB7–Rubpy 0.5 µM vs the added spermidine trihydrochloride concentration (0–0.5 eq. SPER, SD calculated for n ≥ 3). b) Corresponding calibration equation with related error values.*

4. References

[69] A. Koc, R. Khan, D. Tuncel, "'Clicked' Porphyrin-Cucurbituril Conjugate: A New Multifunctional Supramolecular Assembly Based on Triglycosylated Porphyrin and Monopropargyloxycucurbit[7]uril" *Chemistry – A European Journal* **2018**, *24*, 15550–15555.

[70] P. Gruhs, A. Kraft, V. Mahram, M. Neumaier, P. Chakraborty, E. Janz, M. V. Balli, P. Picchetti, L. Prodi, M. Kappes, F. Biedermann, "A Portal-Preorganized CB7-Rubpy Conjugate Enables Motif-Selective Protein Sensing" *Angew. Chem. Int. Ed., **2026**, accepted*. DOI: 10.1002/anie.6821024.

[88] C. Marquez, F. Huang, W. M. Nau, "Cucurbiturils: Molecular Nanocapsules for Time-Resolved Fluorescence-Based Assays" *IEEE Trans. Nanobioscience* **2004**, *3*, 39–45.

[89] N. Dong, J. He, T. Li, A. Peralta, M. R. Avei, M. Ma, A. E. Kaifer, "Synthesis and Binding Properties of Monohydroxycucurbit[7]uril: A Key Derivative for the Functionalization of Cucurbituril Hosts" *J. Org. Chem.* **2018**, *83*, 5467–5473.

[90] J. R. Lakowicz, *Principles of Fluorescence Spectroscopy*, Springer US, Boston, MA, **1999**.

[91] M. Montalti, A. Credi, L. Prodi, M. T. Gandolfi, *Handbook of Photochemistry*, CRC Press, **2006**.

[92] "Edinburgh Instruments - Determining fluorescence lifetimes," can be found under https://www.edinst.com/resource/determining-fluorescence-lifetimes/(accessed 22 October 2025), **n.d.**

[93] A. Sillen, Y. Engelborghs, "The Correct Use of 'Average' Fluorescence Parameters" *Photochem. Photobiol.* **1998**, *67*, 475–486.
